# Loss of the E3 ubiquitin ligases UBR-5 or HECD-1 restores *Caenorhabditis elegans* development in the absence of SWI/SNF function

**DOI:** 10.1073/pnas.2217992120

**Published:** 2023-01-23

**Authors:** Lisa Lampersberger, Francesca Conte, Subhanita Ghosh, Yutong Xiao, Jonathan Price, David Jordan, David Q. Matus, Peter Sarkies, Petra Beli, Eric A. Miska, Nicholas O. Burton

**Affiliations:** ^a^Wellcome Trust/Cancer Research UK Gurdon Institute, University of Cambridge, Cambridge CB2 1QN, UK; ^b^Department of Genetics, University of Cambridge, Cambridge CB2 3EH, UK; ^c^Institute of Molecular Biology, Mainz 55128, Germany; ^d^Medical Research Council London Institute of Medical Sciences, London W12 0NN, UK; ^e^Department of Biochemistry and Cell Biology, Stony Brook University, NY 11790; ^f^Department of Biochemistry, University of Oxford, Oxford OX1 3QU, UK; ^g^Department of Biochemistry, University of Cambridge, Cambridge CB2 1QW, UK; ^h^Wellcome Sanger Institute, Wellcome Trust Genome Campus, Cambridge CB10 1SA, UK; ^i^Department of Epigenetics, Van Andel Research Institute, Grand Rapids, MI 49503

**Keywords:** *C. elegans*, SWI/SNF, UBR-5, development, HECD-1

## Abstract

SWItch/sucrose non-fermenting (SWI/SNF) complexes are a family of chromatin remodelers that are conserved across eukaryotes. Mutations in subunits of SWI/SNF cause a multitude of different developmental disorders in humans, most of which have no current treatment options. Here, we identify an alanine-to-valine–causing mutation in the SWI/SNF subunit *snfc-5* (*SMARCB1* in humans) that prevents embryonic lethality in *Caenorhabditis elegans* nematodes harboring a loss-of-function mutation in the SWI/SNF subunit *swsn-1* (*SMARCC1/2* in humans). Furthermore, we found that the combination of this specific mutation in *snfc-5* and a loss-of-function mutation in either of the E3 ubiquitin ligases *ubr-5* (*UBR5* in humans) or *hecd-1* (*HECTD1* in humans) can restore development to adulthood in *swsn-1* loss-of-function mutants that otherwise die as embryos. Using these mutant models, we established a set of 335 genes that are dysregulated in SWI/SNF mutants that arrest their development embryonically but exhibit near wild-type levels of expression in the presence of suppressor mutations that prevent embryonic lethality, suggesting that SWI/SNF promotes development by regulating some subset of these 335 genes. In addition, we show that SWI/SNF protein levels are reduced in *swsn-1; snfc-5* double mutants and partly restored to wild-type levels in *swsn-1; snfc-5; ubr-5* triple mutants, consistent with a model in which UBR-5 regulates SWI/SNF levels by tagging the complex for proteasomal degradation. Our findings establish a link between two E3 ubiquitin ligases and SWI/SNF function and suggest that UBR5 and HECTD1 could be potential therapeutic targets for the many developmental disorders caused by missense mutations in SWI/SNF subunits.

## Significance

Mutations in subunits of the SWI/SNF chromatin remodeling complex are found in numerous different neurodevelopmental disorders and 25% of all human cancers. Here, we use a model SWI/SNF mutation in *C. elegans* to determine whether SWI/SNF complex abundance is specifically regulated by two highly conserved E3 ubiquitin ligases, and inhibition of these enzymes can restore development to adulthood in SWI/SNF mutants that otherwise die early in development. These substantially advance our understanding of how SWI/SNF abundance is regulated and point toward potential therapeutic targets for the many different pathologies caused by mutations in SWI/SNF subunits.

Chromatin remodelers are adenosine triphosphate (ATP)-powered molecular machines that can directly alter the structure of chromatin by reshuffling or evicting nucleosomes. Therefore, they control the access to DNA elements like enhancers, promoters, and replication origins that need to be exposed to execute essential cellular processes such as transcription, replication, and DNA repair (1). The SWItch/sucrose non-fermenting (SWI/SNF) complexes were the first described chromatin remodelers, originally discovered in genetic screens in *Saccharomyces cerevisiae* in the 1980s (2, 3). Later, SWI/SNF complexes were shown to be conserved across all eukaryotes (4). They consist of 10 to 15 subunits, depending on the organism (5). The human SWI/SNF complex (also known as BAF) is encoded by at least 29 genes, and its core comprises one of the two mutually exclusive catalytic ATPases SMARCA2 or SMARCA4 (6), a heterodimer or homodimer of SMARCC1/2 that acts as a scaffold for other subunits in early complex assembly (7, 8) and SMARCB1, which is important for structural complex integrity (9). Genome-wide mapping of SWI/SNF complexes by ChIP-seq and mass spectrometry analysis of SWI/SNF co-IPs discovered diverse roles for these complexes in gene regulation and numerous interactions with other protein complexes and transcription factors (10). SWI/SNF chromatin remodeling is currently estimated to regulate the expression of approximately 20% of human genes (11). This is especially important in differentiation, where SWI/SNF complexes coordinate proliferation and differentiation decisions by facilitating a balance between activation of lineage-specific genes and suppression of proliferation programs (12, 13).

Complete loss of SWI/SNF function causes embryonic lethality in mice, (14) and even partial loss-of-function mutations in SWI/SNF chromatin remodelers cause developmental disorders such as Coffin–Siris syndrome, Nicolaides–Baraitser syndrome, Kleefstra’s syndrome, Hirschsprung’s disease, and autism in humans (15). The lethality observed in complete loss-of-function mutants complicates our ability to understand the mechanisms by which mutations in SWI/SNF subunits disrupt normal development (15). To circumvent embryonic lethality, studies have focused on studying SWI/SNF function in tissue-specific mouse knockout models (16) or in cell culture where individual gene knockouts are viable (17). However, cell culture models cannot recapitulate the role of SWI/SNF in animal development across tissues, and viable cell culture models are still unlikely to represent complete loss of SWI/SNF function (17).

In contrast to mammalian models, the *Caenorhabditis elegans* genome encodes only a single gene for each of the core SWI/SNF subunits. Deletions of the core subunits *swsn-4* (human *SMARCA2/4*) or *swsn-1* (human *SMARCC1/2*) result in embryonic or larval lethality, respectively, and RNAi-mediated knockdown of *snfc-5* (human *SMARCB1*) similarly results in embryonic lethality (18). However, previous work in *C. elegans* identified a temperature-sensitive *swsn-1* mutation. *swsn-1* temperature-sensitive mutants can develop to adulthood at the permissive temperature of 15 °C but arrest in embryonic development with 100% penetrance at the restrictive temperature of 22.5 °C (19). Mutations in *swsn-1* (in *C. elegans*) and in *SMARCC1/2* (in humans) both cause normal animal development to be disrupted, which suggests that SWI/SNF regulation of development might be a conserved process between nematodes and humans and indicates that the temperature-sensitive *swsn-1* allele in *C. elegans* is a useful model to study SWI/SNF function in development.

Here, we report that a specific mutation in *snfc-5* (human *SMARCB1*) can prevent embryonic lethality and early developmental arrest of *swsn-1* (human *SMARCC1/2*) mutants. In addition, we report that the loss-of-function mutations in either of the genes encoding the E3 ubiquitin ligases UBR-5 or HECD-1 could further restore wild-type development in the *swsn-1* mutant model. Specifically, around 70% of hatched *swsn-1; snfc-5; ubr-5* triple mutants developed to adulthood under conditions, where 100% of *swsn-1* single mutants died as embryos. Using our mutant models, we established a set of 335 genes that were specifically dysregulated in *swsn-1* mutants but exhibited near wild-type expression levels in *swsn-1; snfc-5* double and *swsn-1; snfc-5; ubr-5* triple mutants across three independent RNA-sequencing experiments, suggesting that the dysregulation of some subset of these genes drives the developmental defects observed in *swsn-1* mutants. In addition, using multiple independent approaches, we demonstrated that UBR-5 likely regulates the levels of SWI/SNF subunits to mediate its effects on SWI/SNF function. Our findings provide insights into how defects in SWI/SNF function cause developmental defects and provide evidence suggesting that UBR5 or HECTD1, the human orthologs of UBR-5 and HECD-1, are potential therapeutic targets for developmental defects caused by missense mutations in SWI/SNF subunits.

## Results

### Mutations in *snfc-5*, *ubr-5*, and *hecd-1* Can Prevent Embryonic Lethality and Developmental Arrest in a Mutant Model of Loss of SWI/SNF Function.

**Fig. 1.**
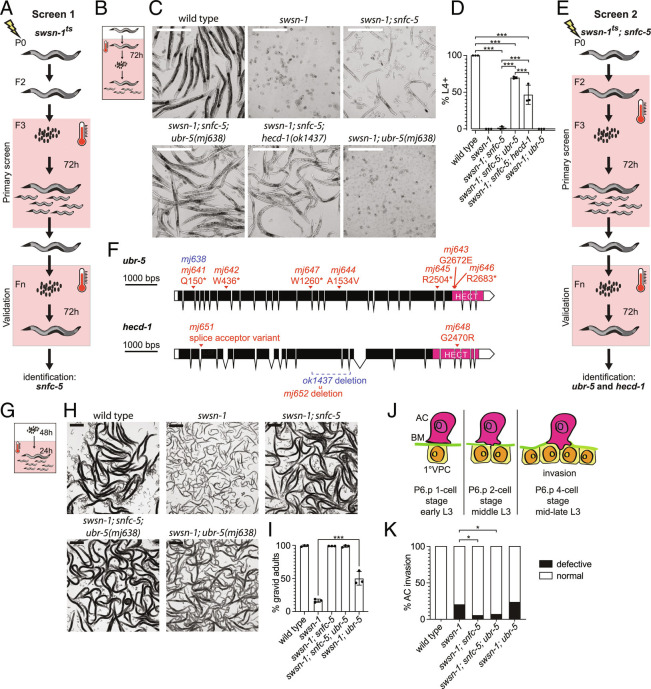
Mutations in *snfc-5*, *ubr-5*, and *hecd-1* can prevent embryonic lethality and developmental arrest of *swsn-1* mutants. (*A*) Schematic of the EMS mutagenesis screen with *swsn-1* temperature-sensitive (ts) mutants. The restrictive temperature of 25 °C is indicated by a pink box and a thermometer. (*B*–*D*) Quantification of *C. elegans* developmental stages after exposing the parental generation to 25 °C for 16 h and collecting and growing embryos at 25 °C for 72 h. (*B*) Schematic of the heat-shock conditions used. (*C*) Representative images of wild-type animals, *swsn-1* single, *swsn-1; snfc-5* double, *swsn-1; snfc-5; ubr-5* triple, *swsn-1; snfc-5; hecd-1* triple and *swsn-1; ubr-5* double mutants. (Scale bar, 500 μm.) (*D*) The percentage of L4 stage or older animals (n = 3 of ≥ 100 animals) is shown. Bar heights represent the mean, and error bars represent SD. *** = Bonferroni corrected Fisher’s exact test *P*-value < 0.0001, calculated using contingency table in *SI Appendix*, Fig. S1*H*. (*E*) Schematic of the EMS mutagenesis screen with *swsn-1; snfc-5* mutants using a longer high-temperature exposure than in *A*. (*F*) Schematic representation of *ubr-5* and *hecd-1* with alleles identified in the second EMS screen (red), CRISPR recreated Q150* *ubr-5* allele (blue) and available *hecd-1* deletion allele *ok1437* (blue). C-terminal HECT domains are indicated in pink. Graphic made using http://wormweb.org/exonintron. (*G*–*I*) Quantification of *C. elegans* developmental stages after embryos were exposed to 22.5 °C for 48 h and 25 °C for 24 h. (*G*) Schematic of the heat-shock conditions used. (*H*) Representative images of wild-type animals, *swsn-1* single, *swsn-1; snfc-5;* double, *swsn-1; snfc-5; ubr-5* triple and *swsn-1; ubr-5* double mutants, larvae in the images of the wild-type, *swsn-1; snfc-5* double and *swsn-1; snfc-1; ubr-5* triple mutants are the offspring of scored animals and were not scored. (Scale bar, 500 μm.) (*I*) Percentage of gravid adults (n = 3 of >100 scored animals). Bar heights represent the mean; error bars represent SD. *** = Bonferroni corrected Fisher’s exact test *P*-value < 0.0001, calculated using the contingency table in *SI Appendix*, Fig. S1*J*. (*J* and *K*) Quantification of AC invasion. (*J*) Schematic of AC invasion, BM = basement membrane, 1° VPC = primary vulval precursor cell. (*K*) Scoring of invasion defects, * = Fisher’s exact test *P*-value < 0.05, calculated using the contingency table in *SI Appendix*, Fig. S1*L*. Alleles used: *swsn-1(ku355), snfc-5(mj633), ubr-5(mj638), hecd-1(ok1437).*

To identify mutations that could compensate for loss of SWI/SNF function, we utilized a temperature-sensitive (ts) loss-of-function allele of the core SWI/SNF subunit *swsn-1* in the model animal *C. elegans* (20)*.* Specifically, we utilized a *swsn-1* temperature-sensitive allele that encodes a P86L substitution mutation in the SWIRM protein domain of SWSN-1 (*SI Appendix*, Fig. S1*A*), which is the exact same mutation as identified by Sawa et al. (19). Of note, 100% of animals homozygous for the *swsn-1* P86L mutation die as embryos when grown at 22.5 °C or arrest their development at early larval stages when exposed to 25 °C after completing embryonic development (19). We mutagenized *swsn-1* mutants with ethyl methanesulfonate (EMS) at a permissive temperature (20 °C) and subjected their F3 offspring as synchronized embryos to the restrictive temperature (25 °C) for 72 h (Fig. 1*A*). We identified five mutant isolates that did not arrest development at early larval stages. By performing whole genome sequencing of these five isolates, we found that one of the recovered isolates carries an additional *swsn-1* substitution mutation (V62I) nearby the original P86L mutation. This mutation is likely an internal suppressor and was not further validated. The remaining four isolates from this screen, which were all from independent pools, all carry an identical A258V substitution mutation in the gene encoding SNFC-5 (*SI Appendix*, Fig. S1*B*), another core subunit of the SWI/SNF complex and homolog of human SMARCB1. We recreated the A258V *snfc-5* mutation by CRISPR-Cas9 gene editing (21) and confirmed that this mutation prevents early larval arrest in *swsn-1* mutants (*SI Appendix*, Fig. S1 *C*–*F*). We conclude that the A258V mutation in *snfc-5* can suppress some of the developmental defects observed in *swsn-1* mutants.

We found that exposure of mutant L4-staged or young adult animals to 25 °C for 16 h prior to collecting the embryos resulted in 100% embryonic lethality for *swsn-1* single mutants and 95% of *swsn-1; snfc-5* double mutants (*SI Appendix*, Fig. S1*G*). Of the few surviving *swsn-1; snfc-5* double mutants, 98% arrested development between L1 and L3 stages (Fig. 1 *B*–*D* and *SI Appendix*, Fig. S1*H*). These findings indicate that this specific mutation in *snfc-5* can suppress the embryonic lethality in a proportion of *swsn-1* mutants but is not sufficient to restore development to adulthood in the animals that do not die as embryos. Therefore, we asked whether we could suppress the developmental defects of *swsn-1; snfc-5* double mutants further by introducing additional mutations and whether this would allow us to identify genetic interactors of the SWI/SNF complex. To test this hypothesis, we performed a second EMS mutagenesis screen with *swsn-1; snfc-5* double mutants using this longer high-temperature period and screened for mutants that could develop to adulthood (Fig. 1*E*). From this screen, we identified and sequenced the genomes of 12 independently isolated mutants that developed to adulthood. We found that two of the mutant isolates have additional mutations in *swsn-1* or *snfc-5* (*SI Appendix*, Fig. S1*I*). These mutations are likely internal suppressor mutations and were not characterized further. Seven of the remaining isolates each carry different predicted loss-of-function mutations in the gene encoding the HECT-type E3 ubiquitin ligase UBR-5 and three have mutations in the HECT-type E3 ubiquitin ligase HECD-1, a paralog of UBR-5 (Fig. 1*F* and *SI Appendix*, Fig. S1*I*). We recreated the UBR-5 Q150* allele, as it was the earliest premature stop mutation we identified (Fig. 1*F*), by CRISPR-Cas9 gene editing (21) (*mj638*). We then crossed *swsn-1; snfc-5* double mutants to *ubr-5(mj638)* mutants and confirmed that this mutation in *ubr-5* restored development to at least the L4 larval stage in approximately 70% of viable animals in the SWI/SNF mutant model (Fig. 1 *B*–*D* and *SI Appendix*, Fig. S1*H*). Moreover, we found that approximately 29% of *swsn-1; snfc-5; ubr-5* triple mutants did not die as embryos, which is a more than fivefold decrease of embryonic lethality compared to *swsn-1; snfc-5* double mutants (*SI Appendix*, Fig. S1*G*). Similarly, we crossed *swsn-1; snfc-5* double mutants with mutants harboring a deletion in *hecd-1* (*ok1437)* and again confirmed that the loss of HECD-1 (introduction of the predicted *hecd-1* null allele) restored development to the L4 larval stage in 46% of animals in our SWI/SNF mutant model (Fig. 1 *B*–*D* and *SI Appendix*, Fig. S1*H*). We conclude that mutations in *ubr-5* and *hecd-1* can restore development to adulthood in *swsn-1; snfc-5* double-mutant animals.

To test whether the loss of UBR-5 (introduction of the predicted *ubr-5* null allele) is sufficient to restore developmental defects in *swsn-1* single mutants even in the absence of the *snfc-5* mutation, we generated *swsn-1; ubr-5* double mutants and assayed their development under different conditions. We found that loss of *ubr-5* alone increased the number of *swsn-1* mutants that developed into gravid adults from 14 to 45% when embryos were grown at room temperature for 48 h, and then shifted to 25 °C for 24 h (Fig. 1 *G*–*I* and *SI Appendix*, Fig. S1*J*) but was not sufficient to restore developmental defects under conditions in which *swsn-1* single mutants died as embryos (Fig. 1 *B*–*D* and *SI Appendix*, Fig. S1*H*). These findings indicate that the mutation in *snfc-5* is required to suppress the embryonic lethality caused by mutation in *swsn-1*, but that the loss of UBR-5 is sufficient to improve development from larvae to gravid adults in *swsn-1* mutants when exposing them to high temperatures for a shorter period at later developmental stages.

Mutations in SWI/SNF subunits cause numerous defects in animal development and physiology. For example, the *swsn-1* mutation also causes defective anchor cell (AC) invasion, a process required for establishing the uterine–vulval connection during larval development critical for adult egg-laying (22) (see the schematic of AC invasion in Fig. 1*J*). To test whether the suppressor mutations we identified specifically suppress the developmental arrest in *swsn-1* mutants or whether they might generally suppress the loss of SWI/SNF function, we assayed AC invasion in wild-type, *swsn-1* mutants*,* and various combinations of double- and triple-mutant animals. We found that *swsn-1* single mutants exhibited defective AC invasion phenotype in 20% (10/50 animals) of animals (Fig. 1*K* and *SI Appendix*, Fig. S1 *K*–*L*), consistent with previously published findings (22). This phenotype was partially rescued in *swsn-1; snfc-5* double (5.2% invasion defects, 4/77 animals) and *swsn-1; snfc-5; ubr-5* triple mutants (6.78% invasion defects, 5/74 animals), but not in *swsn-1; ubr-5* double mutants (23% invasion defects, 7/30 animals) (Fig. 1*K* and *SI Appendix*, Fig. S1 *K*–*L*). These data suggest that the mutation in *snfc-5* can suppress multiple defects observed in *swsn-1* single mutants, but that the loss of UBR-5 specifically suppresses the developmental arrest caused by loss of SWI/SNF function.

### Loss of the UBR-5 Key Catalytic Residue Is Sufficient to Suppress the Developmental Arrest of SWI/SNF Mutants.

**Fig. 2.**
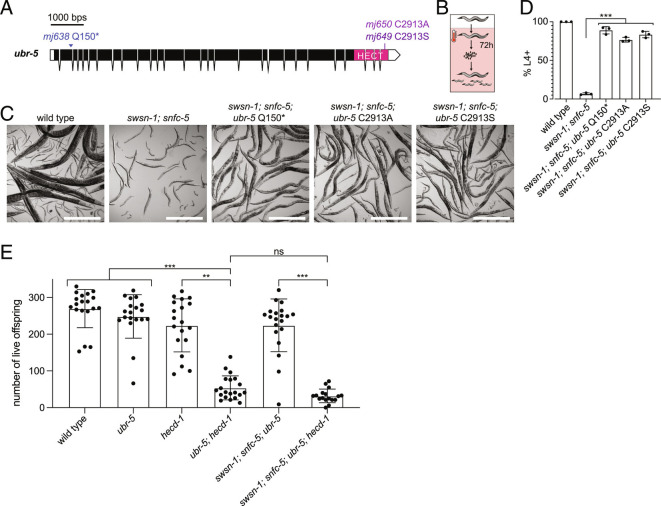
Loss of key catalytic residue of UBR-5 is sufficient for developmental arrest suppression, and the combined loss of UBR-5 and HECD-1 has deleterious effects on *C. elegans* reproduction. (*A*) Schematic representation of *ubr-5* with CRISPR generated Q150*, C2913A, and C2913S substitutions. C-terminal HECT protein domains are indicated in pink. (*B*–*D*) Quantification of *C. elegans* developmental stages after exposing the parental generation to 25 °C for 16 h and collecting and growing embryos at 25 °C for 72 h. (*B*) Schematic of the heat-shock conditions. Restrictive temperature of 25 °C is indicated by a pink box and a thermometer. (*C*) Representative images of wild-type animals (larvae in this image are the offspring of scored animals and were not scored), *swsn-1; snfc-5* double, *swsn-1; snfc-5*; *ubr-5* Q150* triple, *swsn-1; snfc-5; ubr-5* C2913A triple and *swsn-1; snfc-5; ubr-5* C2913S triple mutants. (Scale bar, 500 μm.) (*D*) Percentage of animals L4 larval stage or older (n = 3 of ≥100 scored animals). Bar heights represent the mean, and error bars represent SD. *** = Bonferroni corrected Fisher’s exact test *P*-value < 0.0001, calculated using the contingency table in *SI Appendix*, Fig. S2. (*E*) Number of live offspring of individual wild-type animals, *ubr-5* single, *hecd-1* single, *ubr-5; hecd-1* double, *swsn-1; snfc-5; ubr-5* triple and *swsn-1; snfc-5; ubr-5; hecd-1* quadruple mutant animals. Bar heights represent the mean, and error bars represent SD, Kruskal-Wallis test (H_5_ = 74.76, *P* < 0.001), Dunn’s multiple comparison adjusted *P*-value ** ≤ 0.001, *** ≤ 0.0001, ns = not significant. Alleles used: *swsn-1(ku355), snfc-5(mj633), ubr-5(mj638)* (this *ubr-5* allele was used in *E*)*, ubr-5(mj650), ubr-5(mj649), hecd-1(ok1437).*

HECT-type E3 ubiquitin ligases such as UBR-5 and HECD-1 have a catalytic cysteine within their C-terminal HECT domains (23). Ligation reactions depend on this catalytic cysteine, which forms a thioester-linked intermediate with the ubiquitin before ligating it onto a substrate protein (23). We replaced the catalytic residue of UBR-5 (cysteine 2913) with an alanine or serine by CRISPR-Cas9 gene editing (21) (Fig. 2*A*). The C2913A substitution mutation should prevent the loading of ubiquitin onto UBR-5, whereas the C2913S substitution mutation should still enable the loading but prevent the transfer of ubiquitin onto a substrate (24). We found that substitution of the catalytic cysteine of UBR-5 to alanine or serine in *swsn-1; snfc-5* double mutants resulted in similar suppression of the temperature-sensitive larval arrest as introducing the premature stop *ubr-5* allele (*mj638*) (Fig. 2 *B*–*D* and *SI Appendix*, Fig. S2). These results indicate that inactivation of the catalytic function of UBR-5 is sufficient for the suppression of developmental arrest of *swsn-1; snfc-5* mutants.

### The Combined Loss of UBR-5 and HECD-1 Has Deleterious Effects on C. elegans Reproduction.

Since the loss of either UBR-5 or HECD-1 restored development to adulthood in some of the *swsn-1; snfc-5* double mutants (Fig. 1 *B*–*D* and *SI Appendix*, Fig. S1*H*), we wondered whether the combined loss of both E3 ubiquitin ligases would have an even greater effect. However, when generating *swsn-1; snfc-5; ubr-5; hecd-1* quadruple mutants, we observed that those animals were substantially sicker and had fewer offspring than other mutant combinations. This effect appears to be a synthetic interaction between *ubr-5* and *hecd-1* because we found that *ubr-5; hecd-1* double mutants exhibited a similar phenotype even in the absence of any SWI/SNF subunit mutations. Specifically, we found that the loss of either of the paralogous HECT-type E3 ubiquitin ligases alone did not affect animal reproduction, but the loss of both UBR-5 and HECD-1 resulted in a substantial reduction of live offspring. On average, the *ubr-5; hecd-1* double mutants had about four-times fewer live offspring than either of the *ubr-5* or *hecd-1* single mutants (Fig. 2*E*). The quadruple mutants also had significantly fewer live offspring compared to the *swsn-1; snfc-5; ubr-5* triple mutants and did not have significantly fewer offspring compared to the *ubr-5; hecd-1* double mutants (Fig. 2*E*). Thus, UBR-5 and HECD-1 likely have redundant functions, and loss of one can be compensated by the other protein. To our knowledge, these findings indicate that these two ubiquitin ligases might function redundantly.

### UBR-5 Regulates SWI/SNF Protein Levels.

**Fig. 3.**
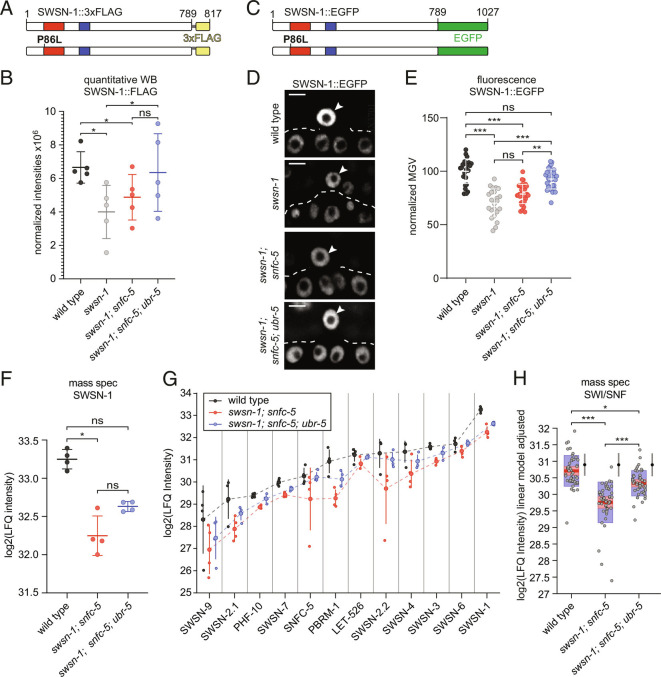
UBR-5 regulates SWI/SNF protein levels. (*A*) In-scale schematic representation of the wild-type and P86L SWSN-1::FLAG proteins. The SWIRM protein domain is shown in red, the SANT protein domain in blue, the glycine linker in gray, and the 3xFLAG-tag in yellow. (*B*) Western blot quantification of SWSN-1::FLAG protein levels in synchronized L1-staged wild-type (*swsn-1::3xflag*), *swsn-1* single-mutant (*swsn-1^ts^::FLAG)swsn-1; snfc-5* double-mutant *(swsn-1^ts^::3xflag; snfc-5)* and *swsn-1; snfc-5; ubr-5* triple-mutant *(swsn-1^ts^::3xflag; snfc-5; ubr-5)* animals (n = 5). The y-axis represents raw integrated densities of SWSN-1::FLAG signals ×10^6^ normalized to total protein signal. RM one-way ANOVA, * = Tukey’s multiple comparisons test adjusted *P*-value < 0.05, ns = not significant. (*C*) In-scale schematic representation of the wild-type and P86L SWSN-1::EGFP proteins. The SWIRM protein domain is shown in red, the SANT protein domain in blue, and the EGFP-tag in green. (*D* and *E*) Quantification of SWSN-1::EGFP intensities in the AC of P6.p 4-cell-staged wild-type (*swsn-1::egfp*)*swsn-1* single-mutant (*swsn-1^ts^::egfp)swsn-1; snfc-5* double-mutant *(swsn-1^ts^::egfp; snfc-5)* and *swsn-1; snfc-5; ubr-5* triple-mutant *(swsn-1^ts^::egfp; snfc-5; ubr-5)* animals (*D*) Representative images of quantified animals; ACs are indicated by white arrowheads. (Scale bar, 5 µm.) (The corresponding DIC images can be found in *SI Appendix*, Fig. S3*A*.) (*E*) SWSN-1::EGFP mean gray values (MGV) of ACs of individually quantified animals, Kruskal-Wallis test (H_3_ = 60.09, *P* < 0.0001), Dunn’s multiple comparison test adjusted *P*-value ** ≤ 0.001, *** ≤ 0.0001, ns = not significant. (*F*) SWSN-1 protein levels in L1-staged wild-type, *swsn-1; snfc-5* double-mutant and *swsn-1; snfc-5 ubr-5* triple-mutant animals determined by label-free proteomics mass spectrometry quantification (n = 4). The y-axis represents log2 LFQ intensities. Kruskal-Wallis test (H_2_ = 8.769, *P* < 0.0012), Dunn’s multiple comparison test adjusted *P*-value * ≤ 0.01, ns = not significant. (*G*) Protein levels of SWI/SNF subunits in synchronized L1-staged wild-type (gray), *swsn-1; snfc-5* double-mutant (red) and *swsn-1; snfc-5 ubr-5* triple-mutant (blue) animals determined by mass spectrometry using LFQ (n = 4). Small dots show protein levels of individual replicates, large dots indicate the mean protein levels, and vertical lines the 95% CIs. The dashed lines connect the mean protein levels of the different subunits for better visualization of the overall trend. The y-axis represents log2 LFQ intensities. (*H*) Box plot representation of the SWI/SNF complex protein levels adjusted for subunit type by multiple linear regression (*SI Appendix*, Fig. S3 *B* and *C*) data of the twelve SWI/SNF subunits from G combined. Red horizontal lines represent the median, light red boxes the 95% CIs of the median, and the blue boxes represent the SD. The y-axis represents log2(LFQ intensities) of each genotype adjusted by subunit based on the linear model. F-test, Bonferroni multiple comparison test adjusted *P*-value * < 0.01, *** < 0.0001. The intervals to the right of the boxplots show the mean (black dots) and SD (blue lines) of 100 randomly chosen sets of 12 proteins. Alleles used: *swsn-1(ku355), swsn-1(syb2756[swsn-1::3xflag]), swsn-1(mj660; syb2756[swsn-1::3xflag]), swsn-1(st12187[swsn-1::egfp]), swsn-1(mj661; st12187[swsn-1::egfp]), snfc-5(mj633), ubr-5(mj638).*

Our findings suggest that the catalytic activity of UBR-5 is involved in suppressing the *swsn-1* mutant developmental arrest (Fig. 2 *B*–*D* and *SI Appendix*, Fig. S2). One of the best-understood roles of ubiquitin ligation to proteins is to tag them for proteasomal degradation (25). The human homologs of UBR-5 and HECD-1, UBR5 and HECTD1 respectively, have both been shown to mediate K48-linked ubiquitination (23), which is a signal for proteasomal degradation (26). A possible and direct link between the SWI/SNF complex and these two ubiquitin ligases could be that the *swsn-1* mutation destabilizes the SWI/SNF complex and that these enzymes ubiquitinate unstable complexes to promote their degradation. In this case, loss of UBR-5 should result in higher levels of SWSN-1, which in turn might explain the observed developmental arrest suppression. To test this hypothesis, we measured SWSN-1 protein levels in wild-type animals, *swsn-1* single, *swsn-1; snfc-5* double and *swsn-1; snfc-5; ubr-5* triple mutants by western blotting. We generated endogenously FLAG-tagged wild-type and mutant versions of SWSN-1 (Fig. 3*A*). We synchronized L1-staged animals by starvation and subsequently fed them and exposed them to the restrictive temperature (25 °C) for 6 h. These conditions were chosen so that all of the mutants would be at closely matched developmental stages. We found that levels of SWSN-1 were on average approximately 40% reduced in *swsn-1* single mutants when compared to wild-type animals, suggesting that the P86L mutation reduces SWSN-1 protein levels. This reduction in SWSN-1 levels was largely restored in *swsn-1; snfc-5; ubr-5* triple mutants (Fig. 3*B*). However, these effects were highly variable, and the levels of SWSN-1 were not statistically significantly different between *swsn-1; snfc-5* double mutants and *swsn-1; snfc-5; ubr-5* triple mutants (Fig. 3*B*) even though we found that most *swsn-1; snfc-5* double mutants arrested their development at early larval stages, while most *swsn-1; snfc-5; ubr-5* triple mutants were able to develop to L4-stages and adulthood (Fig. 1 *B*–*D* and *SI Appendix*, Fig. S1*H*). These results suggest that mutation of *swsn-1* leads to a reduction of SWSN-1 protein levels. Furthermore, UBR-5 potentially has either a small effect on SWSN-1 protein levels or an effect that occurs only in specific cells or at specific developmental stages that is difficult to detect when measuring SWSN-1 levels in whole animals by western blotting.

As an alternative approach to assessing SWSN-1 protein levels at the single cell level in the different mutant animals, we measured SWSN-1::EGFP levels in the AC of mid-late L3-staged animals that had been exposed to the restrictive temperature (25 °C) from the L2-L3 molt/early L3 stage until the P6.p 4-cell stage by confocal fluorescence microscopy. For this purpose, we obtained an available SWSN-1::EGFP strain, in which we introduced the P86L temperature-sensitive mutation (Fig. 3*C*) and crossed it to *snfc-5* and *ubr-5* mutants to generate double and triple mutants. Consistent with the western blot quantifications, we found that SWSN-1 protein levels in the AC were significantly reduced in *swsn-1* single and *swsn-1; snfc-5* double mutants compared to wild-type animals (Fig. 3 *D* and *E* and *SI Appendix*, Fig. S3*A*). In this cell-specific context, we observed a statistically significant increase of SWSN-1 levels in *swsn-1; snfc-5; ubr-5* triple mutants when compared to *swsn-1* single and *swsn-1; snfc-5* double mutants (Fig. 3 *D* and *E* and *SI Appendix*, Fig. S3*A*), indicating that UBR-5 regulates SWSN-1 levels in the AC.

To gain better insight into the role of UBR-5 in regulating the abundance of SWI/SNF subunits, we employed a mass spectrometry approach to look at alterations in protein levels in wild-type animals, *swsn-1; snfc-5* double mutants and *swsn-1; snfc-5; ubr-5* triple mutants. Label-free quantification (LFQ) of whole proteome samples from synchronized L1-staged animals revealed a significant decrease in SWSN-1 protein levels in *swsn-1; snfc-5* double mutants compared to wild-type animals (Fig. 3*F*). Consistently with western blotting results, SWSN-1 levels were partially restored to wild-type levels in *swsn-1; snfc-5; ubr-5* triple mutants (Fig. 3*F*), a trend that was consistent in all detected SWI/SNF subunits (Fig. 3*G*). Notably, every subunit of the complex showed this same pattern; the highest levels were in the wild-type animals, reduced levels in the *swsn-1; snfc-*5 double mutants, and partially recovered levels in the *swsn-1; snfc-5; ubr-5* triple mutants; however, the mean protein abundance of each subunit was different. Therefore, we performed a multiple linear regression analysis to determine the effect of the mutation on the entire complex by regressing out the effect of the different mean expression levels. This analysis revealed that the SWI/SNF complex, as a whole, is significantly depleted in the *swsn-1; snfc-*5 double mutants when compared to wild-type animals and that this depletion is partially rescued in *swsn-1; snfc-5; ubr-5* triple mutants (Fig. 3*H* and *SI Appendix*, Fig. S3 *B* and *C*). The same analysis with 100 randomly chosen sets of twelve proteins was not significant (depicted by the intervals to the right of the boxplots in Fig. 3*H*). *SI Appendix*, Fig. S3 *D* and *E* shows an example of a regression analysis for one set of twelve randomly selected proteins (ARX-6, EXOS-1, CYN-1, NBET-1, EMB-4, ZK1236.5, VPS-29, TFTC-3, MDT-9, TBA-1, HMT-1, and Y39G8B.1). All together, these data indicate that the loss of UBR-5 results in increased protein levels of all SWI/SNF subunits in the *swsn-1; snfc-5* mutant model. Furthermore, these results suggest that UBR-5 likely ubiquitinates the SWI/SNF complex to tag it for proteasomal degradation and that the loss of UBR-5 likely prevents some of the developmental defects exhibited in SWI/SNF mutants by increasing SWI/SNF complex abundance. Our findings that levels of all SWI/SNF subunits of *swsn-1; snfc-5* double mutants are reduced compared to wild-type animals are consistent with previously published mouse data (16).

To determine how UBR-5 and HECD-1 regulate protein levels in animals, we similarly obtained mass spectrometry proteomics data of wild-type animals and *ubr-5* single and *hecd-1* single mutants. When comparing protein levels of SWI/SNF subunits, we found that SWSN-7 and PHF-10 are significantly up-regulated (FDR < 0.05) by 11 and 23%, respectively, in *ubr-5* single mutants compared to wild-type animals (*SI Appendix*, Table S1) and that SWSN-3 is significantly up-regulated (FDR < 0.05) by 10% in *hecd-1* single mutants compared to wild-type animals (*SI Appendix*, Table S2). These data suggest that the steady-state levels of specific individual SWI/SNF subunits could also be regulated by UBR-5 and HECD-1 ubiquitination but that the loss of UBR-5 or HECD-1 does not broadly increase the abundance of all SWI/SNF subunits in wild-type animals.

It remains possible that in addition to regulating SWI/SNF protein levels, UBR-5 also ubiquitinates other proteins and that restoring the levels of these proteins also helps SWI/SNF mutants develop to adulthood. Our proteomics analysis identified twelve significantly up-regulated proteins (fold-change ≥ 1.5: FDR ≤ 0.05) (SKP-1, F08F3.4, FBXA-156, ZK228.4, T19H12.2, F36A2.3, GST-41, PCN-1, HAT-1, HRG-2, HSP-16.1 and HSP-16.49) in *swsn-1; snfc-5; ubr-5* triple mutants when compared to *swsn-1; snfc-5* double mutants (*SI Appendix*, Fig. S3*F*). The collective up-regulation of those 12 proteins in *swsn-1; snfc-5* mutants might contribute to the suppression of observed developmental defects. Furthermore, using the proteomics data of *ubr-5* and *hecd-1* single mutants, we could identify 21 proteins significantly up-regulated in both mutant conditions (*SI Appendix*, Fig. S3*G*), suggesting that the regulation of those proteins might be a redundant function of the two ubiquitin ligases.

### Mutations That Suppress the Embryonic Lethality of swsn-1 Mutants Restore Wild-type Expression of 335 SWI/SNF-Regulated Genes.

**Fig. 4.**
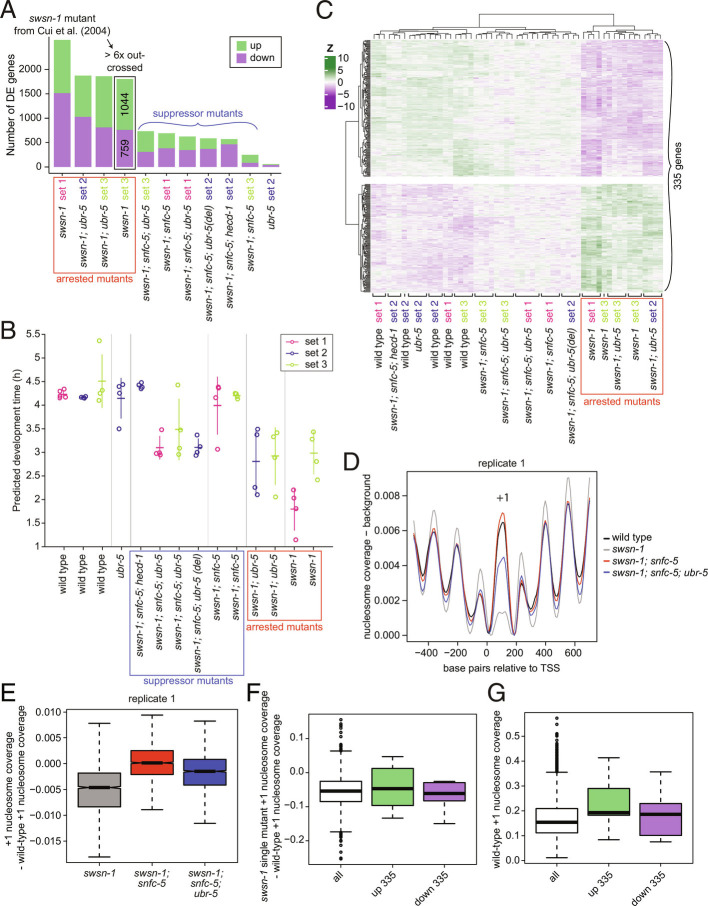
Mutations that suppress the embryonic lethality of *swsn-1* mutants restore wild-type expression of 335 SWI/SNF-regulated genes. (*A–C*) Differential gene expression analysis of three independent RNA-sequencing (RNA-seq) experiments (sets 1 to 3) using synchronized L1-staged wild-type animals and *swsn-1* single, *swsn-1; snfc-5* double, *swsn-1; snfc-5; ubr-5* triple (Q150* and deletion alleles), *swsn-1; snfc-5; hecd-1* triple, *swsn-1; ubr-5* double and *ubr-5* single mutants (n ≥ 4). (*A*) Bar graphs of differentially up- (green) or down-regulated (purple) genes of the different mutants compared to wild-type animals of their respective RNA-seq set. (*B*) Predicted developmental progression of the L1-staged mutants and wild-type animals used for RNA-sequencing estimated by comparing their gene expression profiles to a published wild-type *C. elegans* time course (hourly from 1 to 24 h of larval development) RNA-seq data set (29). Vertical lines represent the 95% CIs and horizontal lines the mean. (*C*) Z-score heatmap of 335 genes consistently differentially expressed (DE) only in *swsn-1* single and *swsn-1; ubr-5* double mutants. (*D*–*G*) Nucleosome coverage analysis of MNase-sequencing (MNase-seq) data using synchronized L1-staged wild-type animals and *swsn-1* single, *swsn-1; snfc-5* double and *swsn-1; snfc-5; ubr-5* triple mutants. (*D*) Nucleosome traces around the TSS of ubiquitous genes. (*E*) Box plots of locus-by-locus +1 nucleosome coverage of ubiquitous genes in the mutants relative to wild-type coverage. Bold horizontal lines represent the median, boxes represent the interquartile range, and whiskers extend to the greatest point ≤1.5 times the interquartile range. (*F*) Box plots of *swsn-1* single-mutant +1 nucleosome coverage minus wild-type +1 nucleosome coverage of all ubiquitous genes (white) and ubiquitous up- (green) and down-regulated (purple) genes from the 335 genes from *B*. Bold horizontal lines represent the median, boxes represent the interquartile range, and whiskers extend to the greatest point ≤1.5 times the interquartile range. Individual data points represent outliers. (*G*) Box plots of the +1 nucleosome coverage in wild-type animals of all ubiquitous genes (white) and ubiquitous up- (green) and down-regulated (purple) genes from the 335 genes from *B*. Bold horizontal lines represent the median, boxes represent the interquartile range, and whiskers extend to the greatest point ≤1.5 times the interquartile range. Individual data points represent outliers. Alleles used: *swsn-1(ku355), snfc-5(mj633), ubr-5(mj638), ubr-5(ok1108)* [used in *swsn-1; snfc-5; ubr-5* triple mutant from RNA-seq set 2, indicated by "(del)"]*, hecd-1(ok1437).*

As chromatin remodelers, the SWI/SNF complexes are thought to control the expression of various genes by enabling or preventing DNA access for the transcription machinery. This involves positioning of nucleosomes to expose promoter and enhancer sequences, thereby enabling the binding of transcription factors and RNA polymerase II (RNA pol II). Similarly, SWI/SNF complexes can facilitate the binding of repressors and disable access to transcription start sites (TSS) (1, 13). Previous studies analyzing the transcriptomes of young adult-staged *swsn-1* mutants reported that between 7.5% (27) and approximately one third (28) of *C. elegans* genes are regulated by the SWI/SNF complex. To identify changes in gene expression that might lead to the developmental defects of *swsn-1* mutants, we conducted three independent RNA-sequencing experiments (sets 1 to 3) using synchronized L1-staged animals (same conditions as for the quantitative western blotting and proteomics experiments). We analyzed gene expression profiles of wild-type animals, *ubr-5* mutants, *swsn-1* mutants*,* and various combinations of double- and triple-mutant animals to investigate how these different mutations affect SWI/SNF-regulated gene expression*.* We found that approximately 8% of *C. elegans* protein-coding genes (1803) were differentially expressed in *swsn-1* single mutants compared to wild-type animals (Fig. 4*A*). The expression changes of most of these genes were restored to wild-type levels in the *swsn-1; snfc-5* double, *swsn-1; snfc-5; ubr-5* triple, and *swsn-1; snfc-5; hecd-1* triple suppressor mutants but not restored in *swsn-1; ubr-5* double mutants (Fig. 4*A*). The gene expression profiles of *swsn-1; ubr-5* double mutants were similar to those of *swsn-1* single mutants (*SI Appendix*, Fig. S4*A*) which is consistent with our previous findings that both of these mutant backgrounds exhibit embryonic lethality under heat-shock conditions where parent animals are exposed to 25 °C for 16 h prior to isolating embryos and exposing them to 25 °C (Fig. 1 *B*–*D*). These data are consistent with our hypothesis that the *snfc-5* A258V mutation generally suppresses the defects caused by the *swsn-1* mutation and that the *ubr-5* mutation alone is not sufficient to suppress defects when animals are exposed to the restrictive temperature at early larval stages. *ubr-5* single mutants had only 62 differentially expressed genes compared to wild-type animals (Fig. 4*A*).

We hypothesized that the suppressor mutants we identified restore developmental defects of *swsn-1* mutants by restoring the expression of developmentally critical genes. However, identifying the precise SWI/SNF-regulated genes that promote animal development is complicated by the asynchronous developmental rate of SWI/SNF mutant animals (see developmental progression estimation for the sequenced animals using an available RNA-seq time-course data set in Fig. 4*B*), which can be slower than that of wild-type animals. To not confound developmentally important genes with genes that are differentially expressed due to developmental staging differences, we compared differentially expressed genes between mutant backgrounds that exhibited early developmental arrest (*swsn-1* single mutants and *swsn-1; ubr-5* double mutants, see *SI Appendix*, Fig. S1*G*) and genetic backgrounds that did not exhibit early developmental arrest (wild-type animals, *swsn-1; snfc-5* double mutants, *swsn-1; snfc-5; ubr-5* triple mutants, *swsn-1; snfc-5; hecd-1* triple mutants, and *ubr-5* single mutants) at the developmentally synchronized L1 larval stage (i.e., all genetic backgrounds were synchronized as L1 stage animals). Furthermore, we performed these RNA-seq experiments in multiple different “sets” (each with four or five biological replicates) and focused only on genes exhibiting consistent expression changes in all experiments. From this filtered analysis, we identified 335 genes (out of the 1,083 genes differentially expressed in *swsn-1* single mutants when compared to wild-type animals) that were consistently differentially expressed in the strongly developmentally arrested *swsn-1* single and *swsn-1; ubr-5* double mutants (Fig. 1 *B*–*D* and *SI Appendix*, Fig. S1 *C*–*H*) but displayed near wild-type gene expression in all mutant backgrounds that did not exhibit the early larval arrest (Fig. 4*C*). We conclude that the dysregulation of some subset of these 335 genes is likely at least partially responsible for driving the early developmental arrest that occurs in *swsn-1* single mutants.

To evaluate the potential direct targets of altered SWI/SNF function on nucleosome positioning at gene promoters, we performed MNase-sequencing of synchronized L1-staged (same conditions as for RNA-seq) wild-type animals, *swsn-1* single, *swsn-1; snfc-5* double and *swsn-1; snfc-5; ubr-5* triple mutants to obtain genome-wide nucleosome coverage profiles. MNase digests in nucleosome-free regions resulting in a higher signal corresponding to regions protected by nucleosomes, which we refer to as higher nucleosome coverage. This can result from increased nucleosome density (i.e., increased probability of a nucleosome being present at all) or a more precisely positioned nucleosome (i.e., the nucleosome is less “fuzzy”) (30). Since only promoters of ubiquitous and germline-specific genes have clear and well-positioned nucleosomes (31) and we sequenced *C. elegans* L1 larvae that do not have extensive germline tissue (32), the MNase-seq analysis was restricted to ubiquitous genes. The clearest difference we observed between wild-type animals and *swsn-1* single mutants was a strongly reduced +1 nucleosome coverage (the first nucleosome downstream of the promoter). The +1 nucleosome coverage in both the *swsn-1; snfc-5* double and *swsn-1; snfc-5; ubr-5* triple suppressor mutants was higher than in *swsn-1* single mutants (Fig. 4*D* and *SI Appendix*, Fig. S4*B*). These findings indicate that SWI/SNF regulates the nucleosomes at the +1 position of ubiquitously expressed genes and that our suppressor mutations can partially restore the defects observed in *swsn-1* single mutants. Notably, the reduction of the +1 nucleosome coverage in *swsn-1* mutants relative to wild-type nucleosome coverage can also be seen comparing across all ubiquitous promoters separately, indicating a consistent difference that affects most ubiquitous promoters (Fig. 4*E* and *SI Appendix*, Fig. S4*C*). The reduced coverage could reflect less well-positioned +1 nucleosomes or reduced nucleosome density at the +1 region. These data suggest that mutation of *swsn-1* affects the chromatin remodeling function of the SWI/SNF complex, which might in turn explain the observed changes in gene expression.

We next asked whether our set of 335 SWI/SNF-regulated genes (defined in Fig. 4*C*) has a distinct nucleosome positioning profile. To do so, we first asked whether genes with altered expression showed larger changes in nucleosome coverage than genes that did not show changes in expression. We compared the +1 nucleosome coverage of all ubiquitous genes to ubiquitously up- (green) and down-regulated (purple) SWI/SNF-regulated genes, which are subsets of the 335 genes. This analysis revealed that the promoters of ubiquitous genes altered in *swsn-1* mutants had a similar reduction in +1 nucleosome coverage relative to wild-type animals (Fig. 4*F*). These results suggest that the coverage of the +1 nucleosome at gene protomers changes for many genes in *swsn-1* single mutants, but this does not always result in changes in their expression.

Interestingly, we found that the subsets of ubiquitously up- (green) and down-regulated (purple) SWI/SNF-dependent genes have a higher nucleosome coverage in wild-type animals when compared to all ubiquitous genes in wild-type animals (Fig. 4*G*). Fig. 4*F* indicates that *swsn-1* mutants also have a higher nucleosome coverage in this subset of genes. These findings confirm that the *swsn-1* mutation studied here does alter proper nucleosome positioning. These findings also show that the 335 SWI/SNF-regulated genes tend to have more well-positioned +1 nucleosomes or a higher nucleosome density than (ubiquitous) genes on average. This could suggest that SWI/SNF-regulated genes depend on a well-positioned +1 nucleosome or on a high nucleosome density for their regulation and that other genes might be more robust to changes in nucleosome coverage.

## Discussion

**Fig. 5.**
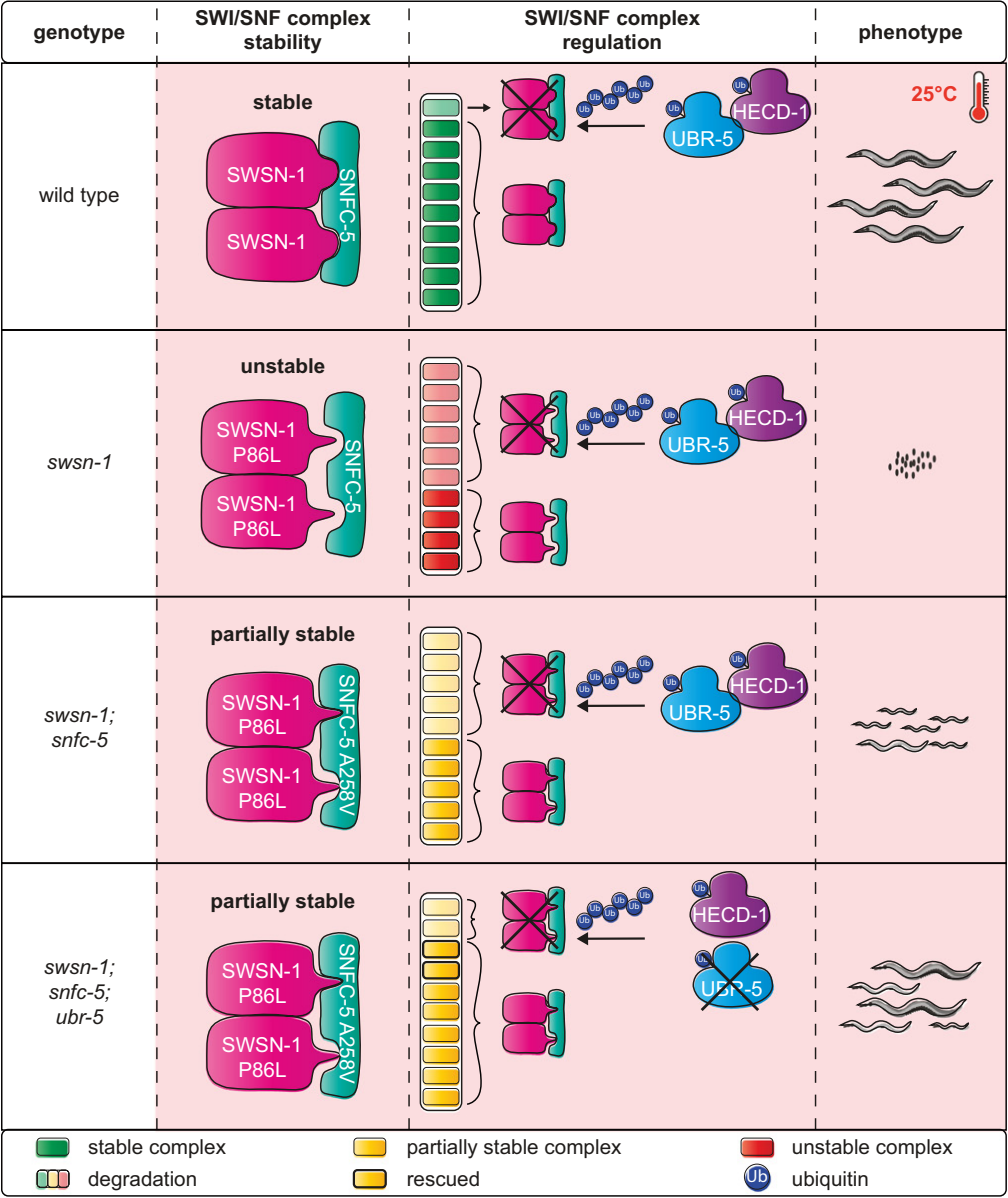
Model of *swsn-1* mutation suppression by *snfc-5* and *ubr-5* mutation. In wild-type animals, a stable SWI/SNF complex can assemble at 25 °C (indicated by a pink box and a thermometer). UBR-5 and HECD-1 ubiquitinate SWI/SNF complexes for proteasomal degradation to maintain their steady-state levels. The *swsn-1* mutation prevents the assembly of the SWI/SNF complex or leads to the assembly of an unstable complex at 25 °C due to structural changes that impair the interaction of SWSN-1 and SNFC-5. Mutant SWI/SNF complexes are more frequently degraded by the proteasome, which is also mediated by UBR-5 and HECD-1 ubiquitination. Additional mutation of *snfc-5* enables the assembly of a partially stable mutant SWI/SNF complex that gets overall less frequently degraded by the proteasome. Loss of UBR-5 leads to an increase of SWI/SNF complex protein levels in *swsn-1; snfc-5* mutants by preventing some of the turnover of the complex by the proteasome.

Here, we identified that the combination of a specific missense mutation in *snfc-5* and the loss of either of two E3 ubiquitin ligases (UBR-5 or HECD-1) can suppress some of the developmental defects caused by a missense mutation in the core SWI/SNF subunit *swsn-1.* UBR-5 and HECD-1 are genetic interactors of the SWI/SNF complex, and our studies revealed a previously unknown functional redundancy between these two ubiquitin ligases in regulating animal development and fertility. In addition, we established that *swsn-1; snfc-5* double mutants have reduced SWI/SNF protein levels and that the loss of UBR-5 can partially restore these levels. Together, these results are consistent with a model in which UBR-5 and HECD-1 promote the degradation of the SWI/SNF complex and that the loss of UBR-5 or HECD-1 can prevent developmental defects from manifesting by increasing SWI/SNF levels (Fig. 5). SWI/SNF levels have been shown to be critical for cell proliferation versus differentiation decisions. Lower levels of SWI/SNF proteins or activity have been associated with cell proliferation both in the M cell lineage (33) and the AC (22) in *C. elegans*. Therefore, by regulating SWI/SNF protein levels, UBR-5 and HECD-1 may be critical for dose-dependent roles of the SWI/SNF complex. In humans, missense mutations in SWI/SNF complex subunits cause a multitude of developmental disorders and cancer and are associated with alcohol use disorders. These include ClinVar annotated missense mutations in the SWIRM domain of human SMARCC1 (His526Pro) and SMARCC2 (Tyr465His, Asn454Asp), analogous to the SWIRM domain mutation in *swsn-1* studied here. Most of these disease-associated mutations in SWI/SNF subunits are heterozygous. Heterozygous mutations identified in developmental disorders are generally dominant, implying that dosage-sensitive processes underlie the roles of SWI/SNF complexes in development (6). Our findings suggest that the inhibition of UBR5 and HECTD1 could be a potential therapeutic strategy to treat developmental disorders caused by dosage-sensitive missense mutations in SWI/SNF complex subunits.

### SWI/SNF Complex Stability.

The human SMARCC1/2 dimer is important in early SWI/SNF complex assembly where it serves as a scaffold to which other subunits bind (7, 8). Studies of SWI/SNF complex assembly found that loss of SMARCC1/2 in mice leads to the dissociation and degradation of the entire complex, presumably because unassembled subunits are less stable (16). In the SWI/SNF mutant *C. elegans* model we used here, a P86L mutation in the SWIRM domain of the *SMARCC1/2* homolog *swsn-1* resulted in embryonic lethality at 25 °C. We rescued the embryonic lethality by introducing a A258V mutation in the RPT2 domain of *snfc-5*, homolog of *SMARCB1*. The SWIRM domains of human SMARCC1/2 and yeast SWI3 dimers directly bind the RPT1 and RPT2 domains of SMARCB1 and SNF5, respectively (8, 34). Based on these data, we propose a model in which the P86L SWSN-1 mutation results in a structural change which prevents stable binding to SNFC-5 and complex assembly. This is supported by the fact that the allele is temperature sensitive, as we expect destabilization to be exacerbated by increased temperature. The A258V SNFC-5 mutation in turn introduces another structural change that allows SNFC-5 to bind mutant SWSN-1 more stably (Fig. 5). The resulting *swsn-1; snfc-5* mutant complex is partially stable, but not as stable as the wild-type assembly, since we find that the levels of all SWI/SNF subunits in *swsn-1; snfc-5* double mutants are reduced compared to wild-type animals (Fig. 3 *G* and *H*). Consistent with this, it has previously been proposed that loss of one SWI/SNF subunit could alter the abundance of the other subunits (10).

We also showed that the levels of all SWI/SNF subunits are partially restored to wild-type levels in *swsn-1; snfc-5; ubr-5* triple mutants (Fig. 3 *G* and *H*). Moreover, even though the loss of either UBR-5 or HECD-1 did not substantially affect SWI/SNF levels in wild-type animals, we found that some individual SWI/SNF subunits were increased by approximately 10 to 20% in *ubr-5* and *hecd-1* single mutants (*SI Appendix*, Tables S1 and S2). This suggests that UBR-5 and HECD-1 can also affect the steady-state levels of wild-type SWI/SNF subunits. Overall, our data are consistent with a model in which UBR-5 (and presumably also HECD-1) directly ubiquitinates either SWSN-1 or another subunit of the SWI/SNF complex to regulate SWI/SNF protein levels. Specifically, our data suggest that *swsn-1* single mutants form an unstable SWI/SNF complex that consequently becomes degraded by the proteasome, resulting in a modest reduction of SWI/SNF protein levels and embryonic lethality. By contrast, *swsn-1; snfc-5* double mutants form a partially stable complex that is less prone to degradation resulting in increased SWI/SNF complex abundance, when compared to *swsn-1* single mutants, and the prevention of embryonic lethality. Finally, the loss of UBR-5 or HECD-1 further increases SWI/SNF complex abundance in *swsn-1; snfc-5* double mutants by preventing some of the turnover of the complex by the proteasome. This ultimately promotes SWI/SNF function and enables *swsn-1; snfc-5; ubr-5* triple mutants to develop to adulthood (Fig. 5).

### Synergistic Functions of UBR-5 and HECD-1.

We identified two paralogous HECT-type E3 ubiquitin ligases as suppressors of SWI/SNF mutation in our screen. Despite the finding that the loss of either of the two enzymes alone appears to promote animal health in *swsn-1; snfc-5* mutants, we found that the combined loss of these two proteins has deleterious effects on animal health. We report a synergistic genetic interaction between these two ubiquitin ligases. These findings suggest that UBR-5 and HECD-1 likely function at least partially redundantly. Our data indicate that the SWI/SNF complex is one likely such common target of UBR-5 and HECD-1; however, our proteomics profiling of *ubr-5* and *hecd-1* single mutants showed only small increases of different individual SWI/SNF subunits. We suspect that due to the potentially redundant functions of UBR-5 and HECD-1, there would be substantially larger changes in SWI/SNF subunit abundance and other protein abundances in *ubr-5; hecd-1* double mutants which cannot properly regulate protein levels via either ubiquitin ligase. These dysregulated protein levels might in turn drive the strong synthetic phenotypes observed in *ubr-5; hecd-1* double mutants.

### UBR-5 Function at Later Developmental Time Points and in Specific Tissues.

Despite our finding that *swsn-1; snfc-5; ubr-5* triple mutants could develop to adulthood, making them better developmental suppressors than the *swsn-1; snfc-5* double mutants, we did not observe an improved rescue of gene expression or of nucleosome coverage in the triple mutants when compared to double mutants. We suspect that the loss of UBR-5 on SWI/SNF-dependent gene expression likely becomes more apparent at later developmental stages. This is supported by our observations that *ubr-5* alone, in the absence of any *snfc-5* mutation, was capable of partially suppressing *swsn-1* single mutants if they survived to a later (post L1) larval stage (Fig. 1 *G*–*I* and *SI Appendix*, Fig. S1*J*). Furthermore, the effect of loss of UBR-5 or HECD-1 could be tissue specific and not detectable in synchronized L1 animals, which is supported by the clear effect of the *ubr-5* mutation on SWSN-1 levels that we observed in the AC of L3-staged animals (Fig. 3 *D* and *E*), compared to synchronized L1s (Fig. 3 *B* and *F*). It has also been shown that certain SWI/SNF accessory subunits have an impact on specific developmental processes, which adds another layoff of regulation to SWI/SNF. For example, PBRM-1 is important for somatic gonadal precursors development, while LET-526 for the distal tip cell (18) and SWSN-2.1 and SWSN-2.2 have distinct roles in embryonic development (35). UBR-5 may have higher activity on certain accessory subunits at different stages in development. Future studies looking at different developmental time points or specific tissues might shed light on how and whether UBR-5 affects the gene expression of SWI/SNF targets during development.

### Evolution of SWI/SNF and Complex Stability.

Interestingly, many animals, including humans, mice, chicken, zebrafish, and fruit flies, all already have a valine at the equivalent position as the A258V substitution in the RPT2 domain of SNFC-5 that we identified as a suppressor in the first mutagenesis screen (*SI Appendix*, Fig. S1*B*). Moreover, one of the mutants isolated in the second mutagenesis screen carries the additional V129I substitution mutation in the SWIRM domain of *swsn-1*, and the human, mouse, and fruit fly SWSN-1 homologs also have an isoleucine at the equivalent position (*SI Appendix*, Fig. S1*A*). These findings suggest that the valine and isoleucine at these specific positions could make the SWI/SNF complex more robust, and these exact substitutions might have played a role in the evolution of SWI/SNF function. For example, these specific substitutions might help animals better adapt to higher temperatures such as those found in warm-blooded animals or even the poikilotherm model animals zebrafish and fruit flies, which develop at approximately 28 °C (36) and 25 °C (37), respectively.

## Materials and Methods

### *C. elegans* Strain Maintenance.

*C. elegans* strains were grown and maintained on nematode growth medium (NGM) agar plates seeded with HB101 bacteria (Caenorhabditis Genetics Center, University of Minnesota, Twin Cities, MN, USA) as food source (38). Strains containing the temperature-sensitive *swsn-1* mutation were routinely kept at 15 °C unless otherwise indicated, and other strains were routinely kept at 20 °C or also at 15 °C to match growth conditions of temperature-sensitive strains. The *C. elegans* strains that were used in this study are derived from the Bristol N2 strain and are listed in *SI Appendix*, Table S3.

### Reagents and Resources.

All *C. elegans* strains including genotypes and reagents used in this study can be found in *SI Appendix*, Table S3. These resources include antibodies, primers, software packages, and accession numbers of data sets.

### Method Details.

#### EMS mutagenesis screening.

Ethyl methanesulfonate (EMS) mutagenesis was performed as described previously by Brenner (38). Larval 4 (L4)-staged *C. elegans* were washed off plates with M9 buffer, collected in 15-mL falcon tubes, and washed three times with M9 buffer. Animals were then incubated with a final concentration of 0.05 M EMS (Sigma-Aldrich) in 4 mL M9 buffer on a rotating wheel for 4 h at room temperature. After four washes with M9 buffer, animals were seeded onto several agar plates and left to recover at 20 °C. Once the F1 offspring of mutagenized animals were gravid adults, F1 animals were bleached to obtain F2 generation animals. L4 and young adult-staged F2 animals were either maintained at 20 °C (first screen) or shifted to 25 °C for approximately 16 h (second screen), and the then gravid adult F2 animals were bleached to obtain F3 generation embryos. The F3 embryos were grown at 25 °C for 3 to 5 d and screened for mutants that developed to adulthood. Individually picked F3 mutant animals with the desired phenotype were recovered at 15 °C.

#### Temperature shift assays.

To assess temperature-sensitive developmental defects of different *swsn-1* mutants and wild-type animals, animals were synchronized by bleaching as described in the section "*Bleaching and Synchronization of C. elegans*." To assess developmental differences between *swsn-1* single and *swsn-1; snfc-5* double mutants, recovered embryos were directly seeded onto NGM agar plates and grown at 25 °C for 48 h (*SI Appendix*, Fig. S1 *D*–*F*). To assess developmental differences between *swsn-1* single and *swsn-1; ubr-5* double mutants, recovered embryos were directly seeded onto NGM agar plates and grown at 22.5 °C for 48 h and 25 °C for 24 h (Fig. 1 *D* and *E* and *SI Appendix*, Fig. S1*K*). To assess developmental differences between *swsn-1; snfc-5* double mutants and *swsn-1; snfc-5; ubr-5* triple mutants, more stringent conditions were used. Recovered embryos were directly seeded onto NGM agar plates, grown at 15 °C until the L4 or young adult stage, and then shifted to 25 °C for 16 h. Gravid adults were bleached, and recovered embryos were seeded onto new NGM agar plates and grown at 25 °C for 72 h (Figs. 1 *A* and *B* and 2 *B* and *C* and *SI Appendix*, Figs. S1*H* and S2).

Animals were classified into categories (L1 or > L1, < gravid adult or gravid adult, and < L4 or L4 +, respectively) and manually counted (three replicates of at least 100 animals per genotype were counted). Subsequently, animals were washed off plates, collected in 1.5-mL tubes with M9 buffer, washed twice in 1 mL M9, pelleted, and M9 buffer-aspirated. To image animals, 18 µL of animals in M9 were deposited onto a glass slide coated with a 2% agarose pad and paralyzed by adding 2 µL 100 mM tetramisole. A coverslip was added and mounted with transparent nail polish. Representative differential interference contrast (DIC) microscopy images of animals were taken with a Leica DM6B microscope (upright microscope with the LAS X imaging system) at 10× magnification and 30-ms exposure time or 4x magnification and 10-ms exposure time.

#### Live offspring counts.

Animals were grown at 20 °C, and twenty L4-staged animals were individualized onto 50 mm NGM agar plates per genotype. On the following day, each egg-laying adult animal was transferred onto a fresh plate. This was repeated approximately every 24 h until the production of embryos ceased. The number of hatched live offspring was counted manually 24 to 72 h after removing the parental adult. Statistical analyses and plotting of data were conducted using GraphPad Prism (v. 9.0.0).

Additional detailed experimental methods for all other techniques can be found in Supporting Information.

### Quantification and Statistical Analysis.

Statistical analyses are described in the individual methods sections. Furthermore, figure legends specify what statistical test and *P*-value cut-off were used to determine statistical significance.

## Supplementary Material

Appendix 01 (PDF)

Dataset S01 (TXT)

## Data Availability

Raw RNA-seq and MNase-seq data are available from NCBI GEO using the accession number GSE218808. Raw proteomics data are available from PRIDE using the accession number PXD037497.
