# Supplementary material for: Loss of the E3 ubiquitin ligases UBR-5 or HECD-1 restores Caenorhabditis elegans development in the absence of SWI/SNF function
Source: Proc Natl Acad Sci U S A. 2023 Jan 23;120(5):e2217992120. doi: 10.1073/pnas.2217992120 (PMC9945973; doi:10.1073/pnas.2217992120)
Supplement: Supplementary file 1 — Appendix 01 (PDF) [file pnas.2217992120.sapp.pdf]

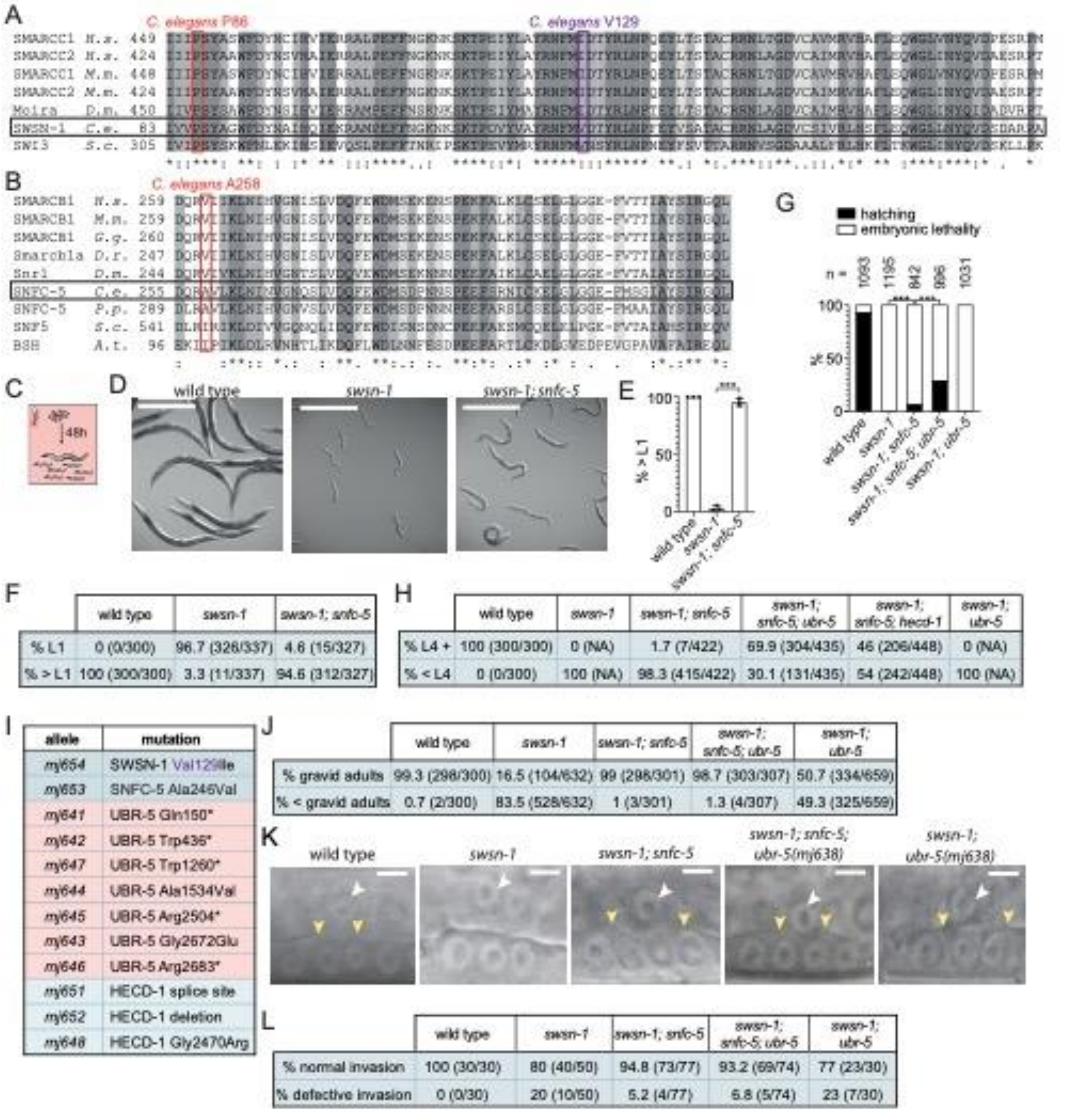

Figure S1: Related to Figure 1. A) Alignments of the SWIRM domains (based on UniProt domain annotations) of human (*H.s.*) SMARCC1 and SMARCC2, mouse (*M.m.*) SMARCC1 and SMARCC2, fruit fly (*D.m.*) Moira, *C. elegans* (*C.e.*) SWSN-1 and yeast (*S.c.*) SWI3 using the UniProt protein alignment tool. '*' and dark grey shading = fully conserved residues, ':' and grey shading = strongly similar residues (> 0.5 in the Gonnet PAM 250 matrix) and '.' and light grey shading = weakly similar residues (< 0.5). The conserved proline mutated in *swsn-1(ku355)* animals is highlighted by a red rectangle. The valine that is mutated in a mutant from the second screen (see J) is highlighted by a purple rectangle. **B)** Alignments of the repeat 2 domain (based on UniProt domain annotation of SMARCB1) of *H.s.* SMARCB1, *M.m.* SMARCB1, chicken (*G.g.*) SMARCB1, zebrafish (*D.r.*) Smarcb1a, *D.m.* Snr1, *C.e.* SNFC-5, *Pristionchus pacificus* (*P.p.*) SNFC-5, *S.c.* SNF5 and Arabidopsis thaliana (*A.t.*) BSH using the UniProt protein alignment tool. Similar and conserved residues are highlighted as described in A. The alanine mutated in *C.e. snfc-5* mutants identified in the first screen is highlight by a red rectangle. **C-F)** Quantification of *C. elegans* developmental stages after exposing embryos to 25°C for 48 hours. (C) Schematic

of the heat-shock conditions, restrictive temperature of 25°C is indicated by a pink box and a thermometer. (D) Representative images of wild-type animals compared to *swsn-1* single and *swsn-1; snfc-5* double mutants, scale bar = 500µm. (E) Percentage of animals older than the larval 1 (L1) stage of >=100 scored animals (n=3). Bar heights represent the mean, error bars represent standard deviation, *** = Bonferroni corrected Fisher's exact test p-value < 0.0001. (F) Contingency table containing combined developmental stage scorings from triplicates of E. This table was used for Fisher's exact test p-value calculations. **G)** Quantification of larval hatching or embryonic lethality at 25°C 24 hours after collecting 842-1195 embryos from wild-type animals, *swsn-1* single, *swsn-1; snfc-5* double; *swsn-1; snfc-5; ubr-5* triple and *swsn-1; ubr-5* double mutants that were grown at 25°C for 16 hours before collecting the embryos. *** = Fisher's exact test p-value < 0.0001. **H)** Contingency table containing combined developmental stage scorings from triplicates of Figure 1D. This table was used for Fisher's exact test p-value calculations. *swsn-1* single and *swsn-1*; *ubr-5* double mutants were not scored, because no larvae hatched. **I)** Table of potential causative mutations of mutants recovered in Figure 1E. **J)** Contingency table containing combined developmental stage scorings from triplicates of Figure 1I. This table was used for Fisher's exact test p-value calculations. **K-L)** Quantification of anchor cell (AC) invasion. (K) Representative images of wild-type animals (normal invasion) compared to *swsn-1* single (defective invasion), *swsn-1; snfc-5* double (normal invasion), *swsn-1; snfc-5; ubr-5* triple (normal invasion) and *swsn-1; ubr-5* double (normal AC invasion, despite defects observed in 23% of animals) mutants, ACs are indicated by white arrowheads, boundaries of breach in the BM are indicated by yellow arrowheads, scale bar = 5µm. (L) Contingency table containing AC invasion scorings of Figure 1K. This table was used for Fisher's exact test p-value calculations. Alleles used: *swsn-1(ku355), snfc-5(mj633), ubr-5(mj638), hecd-1(ok1437).*

	wild type	*swsn-1; snfc-5*	*swsn-1; snfc-5; ubr-5* Q150*	*swsn-1; snfc-5; ubr-5* C2913A	*swsn-1; snfc-5; ubr-5* C2913S
% L4 +	100 (300/300)	6.7 (30/417)	89.3 (410/459)	76.8 (384/500)	83.3 (418/502)
% < L4	0 (0/300)	92.3 (417/447)	10.7 (49/459)	23.2 (116/500)	16.7 (84/502)

Figure S2: Related to Figure 2. Contingency table containing combined developmental stage scorings from triplicates of Figure 2D. This table was used for Fisher's exact test p-value calculations. Alleles used: *swsn-1(ku355), snfc-5(mj633), ubr-5(mj638), ubr-5(mj650), ubr-5(mj649)*

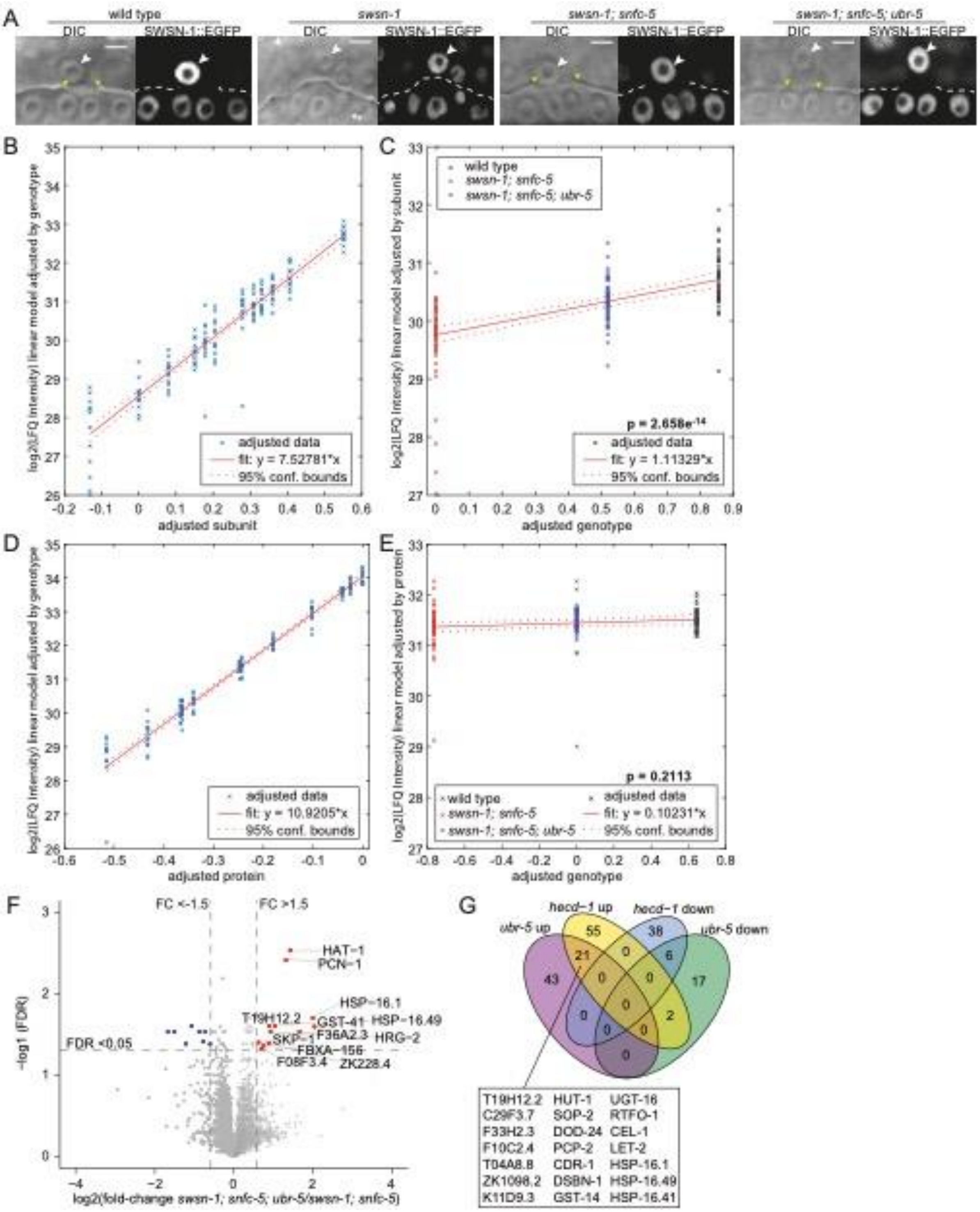

Figure S3: Related to Figure 3. A) Quantification of SWSN-1::EGFP intensities in the AC of P6.p 4-cell-staged wild-type (*swsn-1::egfp*), *swsn-1* single mutant (*swsn-1^{ts}::egfp)*, *swsn-1; snfc-5* double mutant *(swsn-1^{ts}::egfp; snfc-5)* and *swsn-1; snfc-5; ubr-5* triple mutant *(swsn-1^{ts}::egfp; snfc-5; ubr-5)* animals exposed to 25°C from the L2-L3 molt/early L3 stage until the P6.p 4-cell stage. Representative images of quantified animals, ACs are indicated by white arrowheads, boundaries of breach in the BM are indicated by yellow arrowheads, scale bar = 5µm. (The SWSN-1::EGFP images are also displayed in Figure 3D). **B-C)** Linear regression analysis of protein levels of the SWI/SNF subunits (B) Added variable plot for protein subunits displaying adjusted protein levels data (x) from Figure 3G of the three genotypes combined. The x-axis represents scaled distances of subunits to adapt their individual adjusted log2(LFQ intensities) to a linear fit (red line). The y-axis represents log2(LFQ intensities) of the linear model adjusted by genotype. (C) Added variable plot for genotypes displaying adjusted protein levels

data (x) from Figure 3G of the twelve protein subunits combined. The x-axis represents scaled distances of genotypes to adapt their individual adjusted log2(LFQ intensities) to a linear fit (red line). The y-axis represents log2(LFQ intensities) of the linear model adjusted by subunit. ANOVA (p-value = $2.658e^{-14}$) was used to determine that the genotype has an effect on the protein levels of the set of SWI/SNF subunits. **D-E)** Linear regression analysis of protein levels of twelve randomly selected proteins (ARX-6, EXOS-1, CYN-1, NBET-1, EMB-4, ZK1236.5, VPS-29, TFTC-3, MDT-9, TBA-1, HMT-1 and Y39G8B.1) in synchronized L1-staged wild-type, *swsn-1; snfc-5* double mutant and *swsn-1; snfc-5 ubr-5* triple mutant animals determined by label-free proteomics mass spec quantification (n=4). (D) Added variable plot for the twelve proteins displaying adjusted protein levels data of the three genotypes combined. The x-axis represents scaled distances of the proteins to adapt their individual adjusted log2(LFQ intensities) to a linear fit (red line). The y-axis represents log2(LFQ intensities) of the linear model adjusted by genotype. (E) Added variable plot for genotypes displaying adjusted protein levels data of the twelve proteins combined. The x-axis represents scaled distances of genotypes to adapt their individual adjusted log2(LFQ intensities) to a linear fit (red line). The y-axis represents log2(LFQ intensities) of the linear model adjusted by protein. ANOVA (p-value = 0.2113) was used to determine that the genotype has no effect on the levels of this set of twelve randomly selected proteins. **F)** Volcano plot of up- and downregulated proteins in *swsn-1; snfc-5; ubr-5* triple mutants versus *swsn-1; snfc-5* double mutants determined by mass spec using label-free quantification. Fold change < -1.5 or > 1.5, FDR < 0.05. (n=4). **G)** Venn diagram of the overlap between up- and down-regulated proteins in *ubr-5* single and *hecd-1* single mutants compared to wild type animals determined by mass spec using label-free quantification. Fold change < -1.5 or > 1.5, two-sided t-test p-value < 0.05 (n=4). Alleles used: *swsn-1(ku355), swsn-1(syb2756[swsn-1::3xflag]), swsn-1(mj660; syb2756[swsn-1::3xflag]), swsn-1(st12187[swsn-1::egfp]), swsn-1(mj661; st12187[swsn-1::egfp]), snfc-5(mj633), ubr-5(mj638).*

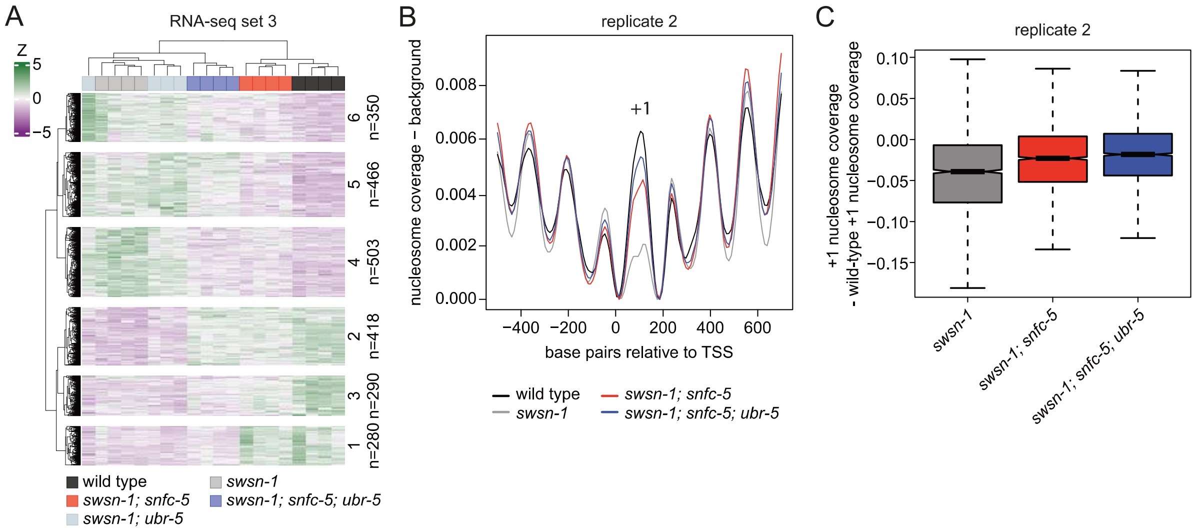

Figure S4: Related to Figure 4. A) Z-score heatmap of the 2307 DEGs differentially expressed between wild type and at least one mutant from RNA-seq set 3 after K-means clustering. **B)** Nucleosome traces around the TSS of ubiquitous genes determined by MNase-seq of synchronized L1-staged wild-type animals and *swsn-1* single, *swsn-1; snfc-5* double and *swsn-1; snfc-5; ubr-5* triple mutants. **C)** Box plots of locus-by-locus + 1 nucleosome coverage of ubiquitous genes determined by MNase-seq in *swsn-1* single, *swsn-1; snfc-5* double and *swsn-1; snfc-5; ubr-5* triple mutants relative to wild-type coverage. Bold horizontal lines represent the median, boxes represent interquartile range and whiskers extend to the greatest point <=1.5 times the interquartile range. Alleles used: *swsn-1(ku355), snfc-5(mj633), ubr-5(mj638), ubr-5(ok1108)* (used in *swsn-1; snfc-5; ubr-5* triple mutant from RNA-seq set 2, indicated by '(del)'), *hecd-1(ok1437).*

Table S1: Fold-change of SWI/SNF protein levels in *ubr-5* mutant versus wild-type. FDR = false discovery rate, FDR < 0.05 highlighted in bold.

**Protein/subunit**	**FC *ubr-5* mutant versus wild-type**	**FDR**
SWSN-1	1.01	0.853
SWSN-4	1.06	0.257
SNFC-5	1.13	0.157
SWSN-2.1	1.15	0.15
SWSN-2.2	1.03	0.329
SWSN-3	1.07	0.205
SWSN-6	0.99	0.901
PBRM-1	1.06	0.656
**SWSN-7**	**1.11**	**0.037**
SWSN-9	1.09	0.49
**PHF-10**	**1.23**	**0.035**
LET-526	0.94	0.303
DPFF-1	1.0	0.982

Table S2: Fold-change of SWI/SNF protein levels in hecd-1 mutant versus wild-type. FDR = false discovery rate, FDR < 0.05 highlighted in bold.

**Protein/subunit**	**FC *hecd-1* mutant versus wild-type**	**FDR**
SWSN-1	1.02	0.829
SWSN-4	0.96	0.833
SNFC-5	1.12	0.291
SWSN-2.1	0.99	0.977
SWSN-2.2	1.08	0.556
**SWSN-3**	**1.1**	**0.043**
SWSN-6	1.06	0.459
PBRM-1	1.06	0.707
SWSN-7	1.14	0.189
SWSN-9	0.99	0.956
PHF-10	1.18	0.142
LET-526	0.93	0.518
DPFF-1	0.86	0.349

Table S3: Reagents and resources used in this study.

**REAGENT or RESOURCE**	**SOURCE**	**IDENTIFIER**
**Antibodies**		
Mouse monoclonal anti-FLAG M2	Sigma Aldrich	RRID: AB_262044
Donkey polyclonal anti-Mouse, IRDye 800CW	Licor	RRID: AB_621847
**Bacterial strains**		
E. coli strain: HB101	Caenorhabditis Genetics Center	HB101
***C. elegans* strains**		
MH2354: *swsn-1(ku355) V*	Cui et al. 2004	MH2354
RW12187: *swsn-1(st12187*[*swsn-1::egfp*]*) V*	Caenorhabditis Genetics Center	RW12187

SX3598: *ubr-5(ok1108) I; snfc-5(mj633) III; swsn-1(ku355) V*	This paper	SX3598
SX3612: *snfc-5(mj633) III; hecd-1(ok1437) IV; swsn-1(ku355) V*	This paper	SX3612
SX3622: *snfc-5(mj633) III; swsn-1(ku355) V*	This paper	SX3622
SX3624: *swsn-1(mj660; syb2756[swsn-1::3xflag]) V*	This paper	SX3624
SX3625: *ubr-5(mj638) I*	This paper	SX3625
SX3627: *ubr-5(mj638) I; snfc-5(mj633) III; swsn-1(ku355) V*	This paper	SX3627
SX3628: *ubr-5(mj638) I; swsn-1(ku355) V*	This paper	SX3628
SX3635: *swsn-1(syb2756[swsn-1::3xflag]) V*	This paper	SX3635
SX3638: *ubr-5(mj638) I; snfc-5(mj633) III; hecd-1(ok1437) IV; swsn-1(ku355) V*	This paper	SX3638
SX3640: *ubr-5(mj649) I; snfc-5(mj633) III; swsn-1(ku355) V*	This paper	SX3640
SX3641: *ubr-5(mj650) I; snfc-5(mj633) III; swsn-1(ku355) V*	This paper	SX3641
SX3644: *ubr-5(mj638) I; hecd-1(ok1437) IV*	This paper	SX3644
SX3645: *ubr-5(mj638) I; snfc-5(mj633) III; swsn-1(mj660; syb2756[swsn-1::3xflag]) V*	This paper	SX3645
SX3653: *snfc-5(mj633) III; swsn-1(mj661; st12187[swsn-1::egfp]) V*	This paper	SX3653
SX3655: *swsn-1(mj661; st12187[swsn-1::egfp]) V*	This paper	SX3655
SX3701: *ubr-5(mj638) I; snfc-5(mj633) III; swsn-1(mj661; st12187[swsn-1::egfp]) V*	This paper	SX3701
SX3714: *swsn-1(ku355) V*	This paper	SX3714
SX3715: *snfc-5(mj633) III; swsn-1(ku355) V*	This paper	SX3715
**Deposited data**		
Proteomics data (deposited to PRIDE)	This paper	PXD037497
RNA-seq data (deposited to GEO)	This paper	GSE218808
MNae-seq data (deposited to GEO)	This paper	GSE218808
**Oligonucleotides**		
gRNA targeting *snfc-5* Ala258: ATTAATATTCAACTTGAGAA	This paper	N/A
gRNA targeting *ubr*-5 Gln150: TGGTGCTGCTGGAGAAGTAG	This paper	N/A
gRNA targeting *ubr-5* Cys2913: GAGGCGGGAAATACACGTGT	This paper	N/A
gRNA targeting *swsn-1* Pro86: TCGAACCAGCCGGCGTATGA	This paper	N/A
Homologous recombination repair template to generate the Ala258Val substitution: GAAGCGCCACCATTGGATGTGAACATTTGTGATCAGAGAGtCGTTCTtAAGTTaAATATcAATGTTGGAAACCAGAGTTTGGTTGATCAATTCGAgtatg	This paper	N/A
Homologous recombination repair template to generate the Gln150* nonsynonymous substitution: GTTTATGCTCGAGCTGGTGCTGCTGGAGAAGTAGAaGTCATTCCATTGAGTGGTGGTATGAACACACTGAGAGCAGCAGCcGGAtAAGCCAAATATCGGAGAGTTATGCTTTCAAACAGg	This paper	N/A

Homologous recombination repair template to generate the Cys2913Ala substitution: CTCAGCAAGCGTCATGCTCCGCCCACAAGAAGATGTATTCCTTCCtACAGCtAACACGgcTATTTCCCGCCTCTATGTACCTGTTTACTCGTCGAAACGTGTCCT	This paper	N/A
Homologous recombination repair template to generate the Cys2913Ser substitution: CTCAGCAAGCGTCATGCTCCGCCCACAAGAAGATGTATTCCTTCCtACAGCtAACACGTcTATTTCCCGCCTCTATGTACCTGTTTACTCGTCGAAACGTGTCC	This paper	N/A
Homologous recombination repair template to generate the Pro86Leu substitution: ACAACTCGCCGAAGGAAACGTCATTGAGCAGACCCACTACATTGTAGTCCtCTCATACGCCGGCTGGTTCGACTATAACGCAATTCATCAAATCGAGAAAC	This paper	N/A
**Software and algorithms**		
Illustrator	Adobe	v. 2021
GraphPad Prism	GraphPad	v. 9.0.0
Fiji	Schindelin et al. 2012	https://fiji.sc/
Excel	Microsoft	v.16.63.1
MATLAB	Mathworks	v. 2022a
Maxquant	Max Plank Institute of Biochemistry	v. 1.5.2.8
Perseus	Max Plank Institute of Biochemistry	v. 1.6.15.0
RStudio	bcorporation	v. 4.1
Trimmomatic	USADELLAB	v. 0.39
BWA Mem	Heng Li	https://github.com/lh3/bwa
Picard tools	Broad Institute	v. 2.27.4
The Genome Analalysis toolkit (GATK)	Broad Institute	v. 4.1.3
Varscan	Daniel C. Koboldt	v. 2.3.8
snpEff	Pablo Cingolani	v. 5.1
bowtie2 aligner	Johns Hopkins University	v.2.2.9
deeptools	Max Plank Institute of Immunology and Epigenetics	v. 3.5.1
MACS2	Tao Liu	v. 2.2.7.1
sortmeRNA	Bonsai Bioinformatics	v. 2.1
salmon	Rob Patro	v. 1.9.0
DESeq2	Michael Love	v. 1.36.0

Table S4: *C. elegans* strains used in the different RNA-seq data sets.

**Strain-name_RNA-seq-set**	**genotype**
N2_third	wild type
SX3627_third	*ubr-5(mj638) I; snfc-5(mj633) III; swsn-1(ku355) V*
SX3628_third	*ubr-5(mj638) I; swsn-1(ku355) V*
SX3714_third	*swsn-1(ku355) V*
SX3715_third	*snfc-5(mj633) III; swsn-1(ku355) V*
N2_second	wild type
SX3625_second	*ubr-5(mj638) I*

SX3598_second	*ubr-5(ok1108) I; snfc-5(mj633) III; swsn-1(ku355) V*
SX3628_second	*ubr-5(mj638) I; swsn-1(ku355) V*
SX3612_second	*snfc-5(mj633) III; hecd-1(ok1437) IV; swsn-1(ku355) V*
N2_first	wild type
MH2354_first	*swsn-1(ku355) V*
SX3627_first	*ubr-5(mj638) I; snfc-5(mj633) III; swsn-1(ku355) V*
SX3622_first	*snfc-5(mj633) III; swsn-1(ku355) V*

Supplemental Materials and Methods

METHOD DETAILS

Bleaching and synchronisation of *C. elegans*

Gravid adult *C. elegans* were washed off NGM plates in M9 buffer (0.6% Na_2HPO_4, 0.3% KH_2PO_4, 0.5% NaCl and 1mM $MgSO_4$) and collected in 15ml Falcon tubes or 1.5ml tubes. Animals in 15ml tubes were pelleted by centrifugation at 800rcf for 1 minute and M9 buffer was aspirated to leave 2ml, animals in 1.5ml tubes were pelleted by centrifugation at 845rcf for 30 seconds and M9 buffer was aspirated to leave 0.5ml. An equal volume of 2x bleaching solution (1M NaOH and 1.5% NaClO - free chlorine) was added and animals were vortexed vigorously for 4-6 minutes to destroy adults and recover embryos. Embryos were washed at least twice in M9 buffer and either directly seeded onto new NGM plates or left to hatch in 5ml M9 rotating on a wheel at 20°C for 24 hours to obtain a synchronized population of L1s.

Collection of synchronized L1-staged *C. elegans*

L1s that hatched after bleaching were counted three times in 10µl drops of M9 buffer to estimate the number of animals present in the 15ml tubes. After 24 hours rotation at 20°C, tubes were spun down at 4000g for 1 minute, M9 buffer aspirated and a certain number of L1s seeded on 9cm NGM agar plates, depending on the assay. At least 30,000 L1s per sample were seeded for Western blotting, 100,000 to 200,000 L1s were seeded for proteomics, 10,000 to 30,000 L1s were seeded for RNA-sequencing and 55,000 to 60,000 L1s were seeded for MNase-sequencing. Subsequently, L1s were grown at 25°C for six hours, a period after which the animals are still at the larval 1 stage of development. L1s were collected into 15ml tubes in M9 buffer with P1000 tips coated in 0.05% TWEEN-20 (Sigma Aldrich) in M9 buffer. Animals were washed three times in 15ml M9 buffer by pelleting animals with 1-minute centrifugations at 4000g and aspiration of buffer.

After the last wash, L1s to be used for Western blotting, proteomics and RNA-sequencing were transferred into 1.5ml tubes with tips coated in 0.05% TWEEN-20 (Sigma Aldrich) in M9 buffer, spun 1 minute at 8000g and remaining M9 buffer was aspirated carefully to leave little buffer

on the pellets. For protein extractions, 100µl pellets of L1s in M9 were snap frozen with liquid nitrogen and stored at -70°C. For RNA extractions, 500µl Trizol reagent (Thermo Fisher Scientific) was added to the pellets and samples stored at -70°C.

For MNase-sequencing, small frozen “worm balls” were generated from the L1 pellets containing little M9 buffer. This was done by dripping small amounts of animal/M9 buffer mix into a cooled ceramic bowl placed on top of dry ice and filled with liquid nitrogen using a glass Pasteur pipette. “Worm balls” were carefully collected into 1.5 ml tubes with a cooled metal spoon and stored at -70°C.

CRISPR-Cas9 gene editing

CRISPR-Cas9 gene editing was performed essentially as described previously[21]. For the injection mixes 0.5µl KCl (0.5M), 0.74µl Hepes pH 7.5 (100mM), 2.5µl tracrRNA (4 µg/µl, Dharmacon), 0.4µl target gene gRNA (4µg/µl, Dharmacon), 0.4µl homologous recombination repair template (1 µg/µl, IDT) and 50ng Pmyo-3::mCherry::unc-54 co-injection plasmid (pCFJ104)[39] were mixed. Then 0.75µl Cas9 (2.5µ/µl, Dharmacon) and DEPC water up to a volume of 10µl were added, mixed, and incubated at 37°C for 15 min. The mix was spun down at max. speed in a tabletop centrifuge, 7.5µl of the mix was transferred into a new tube and micro-injected into the germline young adult staged animals. For the injections, animals were transferred into a drop of halocarbon oil 700 (Sigma Aldrich) on a cover slip with a 2% agarose pad. The animals were straightened in the oil drop using an eyelash pick and positioned so gonad arms could be injected with Femptotip injection capillaries (Eppendorf). An Olympus IX71 microscope equipped with a micromanipulator, FemtoJet injection rig and InjectMan joystick (Eppendorf) was used for the injections. After the injection, M9 buffer was added to the animals to remove the oil and the animals were transferred to individual plates and recovered at 20°C overnight. The offspring of injected animals was then screened for animals expressing the red co-injection plasmid in body wall muscles at a fluorescence microscope. From positive plates, approximately 100 F1 animals were singled onto new plates and genotyped for the introduced allele after they produced F2 offspring. F2 offspring of F1 animals carrying the heterozygous desired allele were singled again and genotyped to obtain a homozygous mutant. The gRNAs and repair templates that were used in this study are listed in Supplemental Table 3.

Embryonic lethality and larval hatching quantification

Synchronized embryos (see section ‘Bleaching and synchronisation of *C. elegans*’) were directly seeded onto NGM agar plates, grown at 15°C until the L4 or young adult stage and then shifted to 25°C for 16 hours. Gravid adults were bleached, recovered embryos were washed 5 times in M9 buffer and counted three times in 10µl drops of M9 buffer to estimate the number of embryos. Approximately 200 embryos were seeded onto 50mm NGM agar plates, counted manually to determine the precise number of embryos seeded and grown at 25°C. After 24 hours, hatched

larvae were manually counted to determine the percentage of larval hatching or embryonic lethality. Five replicates of approximately 200 animals were counted per genotype and numbers combined for Fisher's exact test p-value calculations. Statistical analyses and plotting of data were conducted using GraphPad Prism (v. 9.0.0).

Anchor cell (AC) assays

Assessment of AC invasion

Synchronized animals were grown at 15°C until the L2 molt-early L3 stages, and then shifted to 25°C until the P6.p4-cell stage. AC was defined under differential interference contrast (DIC). An intact barrier under the AC was used to assess invasion. Wild-type invasion was defined as a breach as wide as the basolateral surface of the AC[40]. AC invasion was scored at the P6.p 4-cell stage, when 100% of wild-type animals exhibit a breach in the BM[40].

Live-cell imaging and image quantification

Animals were mounted into a drop of M9 buffer on a 5% Noble agar pad containing approximately 10mM sodium azide anesthetic and topped with a coverslip. Several experiments were scored using epifluorescence visualized on a Zeiss Axiocam MRM camera, also mounted on an upright Zeiss AxioImager A2 and a Plan-Apochromat 100×/1.4 (NA) Oil DIC objective. Microscopy images were obtained on a Hamamatsu Orca EM-CCD camera mounted on an upright Zeiss AxioImager A2 with a Borealis-modified CSU10 Yokagawa spinning disk scan head (Nobska Imaging) using 488nm Vortran lasers in a VersaLase merge and a Plan-Apochromat 100×/1.4 (NA) Oil DIC objective. MetaMorph software (Molecular Devices) was used for microscopy automation. Images were processed using Fiji/ImageJ (v.2.1.0/1.53c)[41]. Expression levels of SWSN-1::EGFP were measured by quantifying the mean grey value of AC nuclei, defined as the somatic gonad cell near the primary vulva. Background subtraction was performed by rolling ball background subtraction (size = 50). Statistical analyses and plotting of data were conducted using GraphPad Prism (v. 9.0.0). Figure legends specify what statistical test and p-value cut-off was used to determine statistical significance.

Preparation of genomic DNA

Starved *C. elegans* were washed of 90mm NGM plates (one plate relatively full of *C. elegans* per strain) in M9 buffer and collected in 15ml tubes. Animals were washed twice in M9 buffer, samples transferred into 1.5ml tubes, pelleted, most of the buffer was removed and pellets stored at -80°C. Animals were lysed with 1ml Cell Lysis Solution (Qiagen) and samples thawed during this process. Five µl Proteinase K (20mg/ml, Thermo Fisher Scientific) was added and incubated at 55°C for approximately 3 hours at 600rpm shaking, until only embryos were left. Lysates were cooled to room temperature and inverted periodically. Then, 5µl RNase A solution (Thermo Fisher Scientific) was added, samples incubated at 37°C shaking for 1 hour and cooled one ice for 3 minutes.

Subsequently, 333µl Protein Precipitation Solution (Qiagen) was added and samples were vortexed vigorously for 20 seconds at high speed. Samples were centrifuged for 10 minutes at 2000rcf and 600µl supernatant transferred into new tubes that were prefilled with 500µl isopropanol and inverted 50 times to mix. After a 2-hour incubation at -20°C, samples were centrifuged at maximum speed for 5 minutes, the supernatant aspirated carefully, and DNA pellet washed with 750µl 75% ethanol by inverting the tubes several times. Samples were centrifuged again at 2000rcf for 3 minutes and supernatant carefully removed with aspirator. Pellets were air dried and resuspended in 35µl water. DNA samples were stored at 4°C.

Whole genome sequencing

Preparation of genomic DNA libraries

Five µl of genomic DNA sample was run on an 1% agarose gel to ensure that DNA fragments were of approximately 10kb sizes. DNA concentrations and A260/A280 ratios were determined using a Nanodrop spectrophotometer (Thermo Fisher Scientific) and concentrations were determined again by Qubit HS dsDNA fluorometric quantification (Thermo Fisher Scientific) according to the manufacturer's instructions. Thirty µl of 100-500ng DNA per sample were prepared. Multiplexed DNA libraries were generated using the Nextera DNA Flex library prep kit (catalog number 20018704, Illumina) and Nextera™ DNA CD Indexes (catalog number 20018707, Illumina) according to the manufacturer's instructions. Libraries were quantified again by Qubit (Thermo Fisher Scientific) and quality control was performed by TapeStation run using a D1000 ScreenTape (Agilent). Samples were sequenced on a HISeq 1500 machine (Illumina) in paired end mode with 50bp read length.

Data processing and analysis

Raw DNA sequencing reads were trimmed for low quality and adapters using Trimmomatic[42] (Version 0.39, parameters: ILLUMINACLIP: TruSeq3-SE.fa:2:30:10 SLIDINGWINDOW:4:20 MINLEN:20). Clean reads were aligned to the *C. elegans* WBCel235 reference genome using BWA Mem[43]. PCR duplicated reads were removed using Picard tools. The Genome Analalysis toolkit (GATK)[44] was then used to re-align the reads across variants using RealignerTargetCreator and IndelRealigner. Varscan[45] was used to identify differences (SNPs and Indels) between the samples and the reference genome. Finally, snpEff[46] was used to annotate the variants.

RNA extraction and RNA-sequencing

Five freeze-thaw cycles were performed with Trizol samples that were stored at -70°C (see section 'Collection of synchronized L1-staged *C. elegans*'), by thawing samples in a warm or hot water bath and immediately refreezing samples in liquid nitrogen. RNA extraction and transcriptome sequencing was performed by BGI Tech Solutions (HongKong) Co.Limited. RNA samples were

sequenced using the BGI DNBSEQ Eukaryotic Strand-specific Transcriptome Resequencing product.

RNA-sequencing analysis

Strains used in the three RNA-seq data sets are listed in Table S4. RNA sequencing reads from the three experimental sets and the developmental time-course RNA-seq data[32] were treated in the same way. Raw reads were trimmed for adapters, low quality sequences and short reads with Trimmomatic[42] (Version 0.39, parameters: ILLUMINACLIP: TruSeq3-SE.fa:2:30:10 SLIDINGWINDOW:4:20 MINLEN:20). Remaining ribosomal RNA was removed with sortmeRNA[47] (Version 2.1, default parameters), clean reads were aligned to the *C. elegans* WBCel235 reference genome and gene counts quantified with salmon[48] (Version 1.9.0, parameters: --gcBias, --sepBias, -l A). Differentially expressed genes were then identified with DESeq2 (FDR < 0.01, |Log2FC| > 1)[49], whereby pairwise comparisons were made between each mutant sample and the wild-type sample (N2) from the corresponding set. A combined DESeq2 results table containing each of these pairwise comparisons can be found in Table S5. All visualisation was produced with the programming language R and the ggplot2 library.

Developmental progression estimation

Developmental progression was estimated by comparing gene expression data to a gene expression time-course obtained from Meeuse et al.[32] where hourly time points were taken from arrested and synchronized L1 larval animals and sequenced. Our data also came from arrested synchronized L1 animals that had been recovered for 6 hours. First, both our data and the Meeuse et al. data were aligned to the same reference to obtain read counts. Then our gene expression profile was compared to the time course profile by first using PCA to embed the Meeuse data alone. Using the eigenvectors obtained from this embedding, our data was embedded into the same space. From this the distance from each point to the piecewise linear curve determined by the time course data was computed. Finally, the time from the nearest two time points was interpolated and used as the estimated developmental time of our samples (see Figure S4B). Data was plotted using a custom function written in MATLAB for plotting raw data points with random jitter, along with the mean and 95% confidence interval.

MNase-sequencing

In vivo MNase digestion

In vivo MNAse digestion was performed on purified nuclei isolated from *C. elegans* (wild type, *swsn-1* single, *swsn-1; snfc-5* double and *swsn-1; snfc-5; ubr-5* triple mutant) L1 frozen "worm balls" (see section 'Collection of synchronized L1-staged *C. elegans*'). Prior to nuclei harvest, frozen "worm balls" containing 55,000 to 60,000 L1s for each strain were individually homogenized in a liquid nitrogen cryo cup. Nuclei purification was optimized using a nuclei pure prep nuclei

isolation kit (NUC-201, Sigma Aldrich). Briefly, 400µl of ice-cold Lysis Solution containing DTT and Triton X-100 was added to each homogenized sample, vortexed and incubated on ice for 5 minutes. Cell lysis and nuclei morphology was determined by microscopic examination to ensure proper homogenization by taking 2µl sample. Nuclei were purified by centrifugation through 1.8 M sucrose cushion solution according to the manufacturer's protocol. Briefly, 900µl of cold 1.8 M sucrose cushion solution was added to each 500µl lysate sample on ice and gently mixed. For each sample preparation, 500µl of ice cold 1.8 M sucrose cushion solution was added to the bottom of a fresh 2ml Eppendorf tube on ice. A total of 1.4ml of lysate solution from the previous step was then slowly layered on top of the 500µl of sucrose cushion solution and set for bench top centrifugation for 45 minutes at 13,000 rpm at 4°C. Supernatant (cytoplasm and cell debris) and the clear sucrose cushion layers were removed without disturbing the pellet of purified nuclei at the bottom of each tube. Nuclei pellet was vortexed briefly and resuspended with 500µl cold Nuclei PURE Storage Buffer. Nuclei pellet were collected by centrifugation at 500g for 5 minutes at 4°C, resuspended again with 50µl cold Nuclei PURE Storage Buffer and vortexed again to completely suspend the nuclei pellet. Qubit HS dsDNA quantification was performed on the purified nuclei to estimate the nucleic acid content. Purified Nuclei of 500ng/reaction were digested with MNAse (M0247S, NEB). The concentration of MNase was titrated for each reaction to obtain mononucleosomes. 250U/ml resulted in mononucleosomes for replicate 1. A concentration of 200U/ml resulted in mononucleosomes for replicate 2. Digestion was for 15 minutes at 37°C. MNase digestion was terminated by adding stop solution containing 3% SDS, 20mM EDTA (final concentrations). Mononuclesomes were treated with Proteinase K (Invitrogen) and incubated at 65°C for 1 hour followed by DNA purification using Zymo clean and Concentrator. Mononucleosome bands were confirmed by D5000 ScreenTape in an Agilent 2200 TapeStation system.

For library preparation, DNA was measured again with Qubit dsDNA HS Assay Kit and 65ng of mononucleosomal purified DNA was used for NEBNext Ultra II DNA Library Prep Kit (Illumina) with size selection. Nucleosomal DNA libraries were pooled in groups of 8 per lane and quantified with by TapeStation (Agilent 2200 TapeStation system) run using a D1000 ScreenTape (Agilent). Libraries were sequenced on an NextSeq 2000 machine (Illumina) in paired end mode with 60bp read length.

MNAse-seq data processing and analysis

The sequenced paired-end reads were mapped to the *C. elegans* (ce11) genome using bowtie2 aligner v.2.2.9[50] with default parameters. The resulting bam files were converted to bigwig tracks using deeptools bamCoverage[51] with parameters -bs 1 --extendReads 148 –normalizeUsing RPGC --effectiveGenomeSize 98259998. Additional paraments for bamCoverage was comprised of --MNase --minFragmentLength 100 --maxFragmentLength 200 --Offset 1 2 and --smoothLength 30. Peak calling was performed with MACS2[52] with default parameters and reads were shifted by

75bp to cover the nucleosome dyad and extended to 150bp. Deeptools computeMatrix reference-point was used to compute the nucleosome density across a window spanning the TSS (500bp upstream to 700bp downstream). The TSS positions were extracted from Serizay et al.[30], as the midpoint of the 300bp regions annotated as ”promoter”. Assignments of each promoter to Ubiquitous, Germline or Somatic was taken from these annotations. The resulting files were read in to R using the read.table function. To produce metagene plots spanning the region surrounding the TSS, the signal at 10bp intervals across the region was averaged across all promoters within the categories above. The ”background” intensity was defined as the minimum value across each trace and was subtracted from each position across the window. A clear signal indicating the expected nucleosome density was only observed for Ubiquitous genes, as expected since somatic promoters do not show such well-defined nucleosome densities[30] and L1s do not have extensive germline tissue[31]. To compare the intensity of the +1 nucleosome across all ubiquitous promoters, the signal spanning from the TSS to 180bp downstream was extracted for each promoter and the signal for wild type subtracted from the mutant signal. To link changes in gene expression to changes in nucleosome density at promoters, the coordinates of the genes were extended by 500bp upstream and intersected with the 300bp promoter regions using bedtools slop and intersect respectively. Nine of the upregulated and 14 of the downregulated genes overlapped with ubiquitous promoters and the +1 signal was extracted and compared across strains.

Generation of C. *elegans* protein extracts

Frozen L1 *C. elegans*/M9 pellets (see section ‘Collection of synchronized L1-staged *C. elegans*’) were thawed on ice, and 25µl DEPC H_2O and 125µl of 2x protein lysis buffer (50mM Tris (pH 7.5), 300mM NaCl, 3mM $MgCl_2$, 2mM dithiothreitol (DTT), 1% Igepal, complete proteinase inhibitor cocktail (Roche)) were added to the 100µl L1/M9 mix. Samples were transferred into 2ml Beadbug™ tubes prefilled with 0.5mm zirconium beads (Merck Life Science) L1s were then broken up to extract proteins using a Beadbug homogenizer (Merck Life Science) at the highest setting for 90 seconds three times at 4°C. After every homogenisation step with the Beadbug, samples were centrifuged at maximum speed at 4°C for 2 minutes followed by 30 seconds at room temperature to remove bubbles and to check the lysis of the animals. Lysates were transferred into 1.5ml protein lobind tubes (Eppendorf), centrifuged at maximum speed for at least 30 minutes at 4°C and supernatants were transferred into new 1.5ml tubes. Subsequently, protein concentrations were determined using the Pierce BCA protein assay kit (Thermos Fisher Scientific) according to the manufacturer’s instructions and measuring the absorbance at 562nm with a spectrophotometer (Microplate Absorbance Reader, Hidex). Relative protein concentrations of samples were calculated using a bovine serum albumin (BSA) standard curve. Protein extracts were snap-frozen in liquid nitrogen and stored at stored at -70°C or used directly.

SDS-PAGE

For sodium dodecyl sulphate polyacrylamide gel electrophoresis (SDS-PAGE), 25-30µg of *C. elegans* L1 protein extracts were supplemented with 10x NuPAGE Sample Reducing Agent and 4x LDS sample buffer (Thermo Fisher Scientific) to a final 1x concentration and denatured at 95°C for 5 minutes. Proteins were resolved alongside a protein ladder (PageRuler Plus Pre-stained Protein Ladder, Thermo Fisher Scientific) on NuPAGE 4-12% BisTris gradient gels using NuPAGE MOPS SDS running buffer (Thermo Fisher Scientific). Gel electrophoresis was routinely performed at 150V for approximately 1 hour and 15 minutes in a XCell SureLock electrophoresis system (Thermo Fisher Scientific) connected to a Bio-Rad PowerPac (Bio-Rad).

Western blotting

Separated proteins were wet transferred onto 0.45mm pore-sized PVDF membranes (Immobilon®-FL PVDF membrane, Millipore) for 2 hours at 250mA and 4°C using cold transfer buffer (1.5x NuPAGE transfer buffer (Thermo Fisher Scientific), 10% methanol, 250µl 20% SDS/L) in a Mini Trans-Blot Cell (Bio-Rad). After the transfer, PVDF membranes were washed in water for 5 minutes, followed by a 2-minute shaking wash in methanol and another 5-minute wash in water. Membranes were air dried, rehydrated in methanol and TBST and incubated with Revert 700 Total Protein Stain (Licor) according to the manufacturer's instructions. To obtain total protein signals for protein signal normalisation, membranes were imaged in a Licor Odyssey Imager in the 700nm channel.

Subsequently, membranes were blocked in 5% (w/v) milk in TBS-0.1% Tween 20 (TBST) for 1 hour at room temperature shaking, followed by an overnight 4°C rotating incubation with the mouse anti-FLAG M2 primary antibody (Simga Aldrich) at 1:1000 dilution in 5% milk. After three 5-minute shaking washes in TBST, membranes were incubated with a secondary IRDye 800CW anti-Mouse antibody (Licor) at 1:10,000 dilution in 5% milk in TBST for one hour at room temperature. Finally, membranes were washed again three times in TBST for 5 minutes shaking and imaged in the 800nm channel of the Licor Odyssey Imager to obtain SWSN-1::FLAG signals.

Quantification of SWSN-1::FLAG protein levels

To quantify protein levels obtained by Western blotting, detected SWSN-1::FLAG signals were normalized to total protein signals. For this purpose, we subtracted the background of the 800nm SWSN-1::FLAG images by creating background images using the morphological opening (imopen) function with a disk shaped structuring element of radius 75 pixels and taking the absolute difference between the image and this background using MATLAB (v2021a Mathworks, Natick, MA). In Fiji (version 2.1.0/1.53c)[41], SWSN-1::FLAG signals of the background subtracted 800nm images were measured by recording the raw integrated densities within a rectangle drawn around the detected SWSN-1 band. This rectangle was moved across the image to record the SWSN-1::FLAG signals of every sample without changing its size. Furthermore, total protein signals of the 700nm images were recorded in Fiji, to determine the relative amounts of proteins

that were loaded into each lane of the polyacrylamide gel and transferred onto the PVDF membranes. To do this, the raw total protein stain 700nm images were rotated to straighten the lanes if necessary. Then, a line with a width of 10 was drawn from top to bottom through one of the protein lanes covering all proteins. This line was moved across all sample lanes of the image and the line profile data for every lane was recorded from a central position of each lane. In excel, the wild-type SWSN-1::FLAG line profile data was plotted against each *swsn-1* single, *swsn-1; snfc-5* double and *swsn-1; snfc-5 ubr-5* triple mutant SWSN-1::FLAG line profiles and linear trendlines were added. The line profiles have intensity peaks where the bands occur in the total protein Western blot. The relative total protein levels were computed by comparing the intensity of each of these peaks in each of the lanes and determining a multiplicative factor that relates their relative intensities. To align the peaks to compute this factor, we performed a lag cross correlation correction to test if the collected line profiles matched well together or if shifting the individual data points up or down by a certain lag increased the correlation between the wild-type and mutant line profiles. We performed the lag cross correlation correction using xcorr function in MATLAB. The slope of linear trendlines for the best correlated lag were calculated and used as dilution factors to normalize the recorded mutant SWSN-1::FLAG signals to; the dilution factor for wild-type SWSN-1::FLAG is 1. The total protein normalized raw integrated densities of SWSN-1::FLAG signals of five independent quantitative Western blots were plotted in Prism (version 9.0.0) and a repeated measurements one-way ANOVA was performed to determine if samples were from the same distribution. After rejecting the null hypothesis, Tukey's multiple comparison test was used to compare FLAG signals between all the conditions.

Label-free proteomics and mass spec

Label-free proteomics

For proteome analysis, protein lysates (see section 'Generation of *C. elegans* protein extracts') were supplemented with LDS Sample Buffer (NuPAGE LDS sample buffer, Thermo Fisher Scientific) with 1 mM dithiothreitol (DTT). Samples were heated at 70°C for 10 min, alkylated by addition of 5.5 mM chloroacetamide, and 50 µg were loaded onto 4-12% gradient Bis–Tris gels. Proteins were separated by SDS–PAGE, stained using the Colloidal Blue Staining Kit (Life Technologies) and in-gel digested using trypsin. Peptides were extracted from gel and desalted on reversed-phase C18 StageTips[53].

Mass spec analysis

Peptides were analyzed on a quadrupole Orbitrap mass spectrometer (Exploris 480, Thermo Scientific) equipped with a UHPLC system (EASY-nLC 1200, Thermo Scientific) as described[54,55]. The mass spectrometer was operated in data-dependent mode, automatically switching between MS and MS2 acquisition. Survey full scan MS spectra (m/z 300–1,700) were acquired in the Orbitrap. The 15 most intense ions were sequentially isolated and fragmented by higher energy C-

trap dissociation (HCD)[56]. An ion selection threshold of 5,000 was used. Peptides with unassigned charge states, as well as with charge states < +2, were excluded from fragmentation. Fragment spectra were acquired in the Orbitrap mass analyzer.

Peptide identification

Raw data files were analyzed using MaxQuant software (development version 1.5.2.8)[57]. Parent ion and MS2 spectra were searched against a database containing 28420 *C.elegans* protein sequences obtained from WormBase (WS269 release), as well as against 4190 proteins in the Ensembl Bacteria *E. coli* REL606 database using the Andromeda search engine[58]. Spectra were searched with a mass tolerance of 6 ppm in MS mode, 20 ppm in HCD MS2 mode, strict trypsin specificity and allowing up to two miscleavages. Cysteine carbamidomethylation was set as a fixed modification, whilst N-terminal acetylation and oxidation were set as variable modifications. The dataset was filtered based on posterior error probability (PEP) to arrive at a FDR < 1% estimated using a target-decoy approach[59]. LFQ quantification was performed in the MaxQuant software using at least two LFQ ratio counts; the fast LFQ and the match between run options were turned on.

Data processing

Processed data from MaxQuant was analyzed in Perseus (version 1.6.15.0)[60] and visualized with RStudio (v. 4.1). Proteins or peptides flagged as “reverse”, “only identified by site” or “potential contaminant” were excluded from downstream analysis. Only proteins identified by no less than two peptides and at least one unique peptide were used in downstream analysis. Proteins mapped to the *E.coli* protein database were discarded. This dataset was further filtered so that proteins identified in at least two out of four replicates for each condition were kept. Missing values were imputed using random values from a Gaussian distribution using the default parameters in Perseus. P-values were calculated with a standard two-sided t-test and Benjamini-Hochberg correction was used for FDR calculation. Data visualization was performed using in-house R scripts and with existing libraries (ggplot2-v 3.3.5, ggrepel-v 0.9.1, RColorBrewer-v 1.1.2, VennDiagram-v 1.7.1) (see Figure S3F-G). Statistical analyses and plotting of data for Figure 3F were conducted using GraphPad Prism (v. 9.0.0).

Linear regression analysis of SWI/SNF protein levels

To determine the effect of the *swsn-1; snfc-5* double and *swsn-1; snfc-5; ubr-5* triple mutants on the protein levels of the SWI/SNF complex as a whole, a linear regression model was fit using the fitlm function in MATLAB (v2021a, Natick MA). The model was fit using both the subunit/protein identity and the genetic background as independent variables. As these are non-numeric variables, the fitlm procedure assigns an arbitrary value to obtain the best linear fit (see Figure S4B-E). fitlm uses an ANOVA to return an f-statistic which tests the null hypothesis that the model is not different from a null model, and also provides a t-statistic for the null hypothesis that each coefficient is

different from 0. This procedure then estimates the best fit coefficient for each subunit (this can be thought of as the correction factor for the varying subunit expression levels) and regresses this out, leaving the effect of the genetic background (see Figure 3H). For reference, randomly selected sets of 12 proteins were analyzed in the same way, first each set was fit with a linear regression model and then the mean adjusted LFQ intensity was calculated for each of the genetic backgrounds. 100 such bootstrap samples were generated and their adjusted mean expression levels are shown as black dots with grey lines indicating the standard deviation in Figure 3H. These data were plotted using a custom function in MATLAB to plot the adjusted response.

Supplemental References:

39. Frøkjær-Jensen, C., Wayne Davis, M., Hopkins, C. E., Newman, B. J., Thummel, J. M., Olesen, S. P., Grunnet, M. & Jorgensen, E. M. Single-copy insertion of transgenes in *Caenorhabditis elegans. Nat. Genet.* **40,** 1375–1383 (2008).

40. Sherwood, D. R. & Sternberg, P. W. Anchor cell invasion into the vulval epithelium in *C. elegans. Dev. Cell* **5,** 21–31 (2003).

41. Schindelin, J., Arganda-Carreras, I., Frise, E., Kaynig, V., Longair, M., Pietzsch, T., Preibisch, S., Rueden, C., Saalfeld, S., Schmid, B., Tinevez, J. Y., White, D. J., Hartenstein, V., Eliceiri, K., Tomancak, P. & Cardona, A. Fiji: an open-source platform for biological-image analysis. *Nat. Methods* **9,** 676–682 (2012).

42. Bolger, A. M., Lohse, M. & Usadel, B. Trimmomatic: a flexible trimmer for Illumina sequence data. *Bioinformatics* **30,** 2114–2120 (2014).

43. Li, H. Aligning sequence reads, clone sequences and assembly contigs with BWA-MEM. (2013). Accessed August 20, 2022. doi:10.48550/arxiv.1303.3997

44. Poplin, R., Ruano-Rubio, V., DePristo, M. A., Fennell, T. J., Carneiro, M. O., Auwera, G. A. Van der, Kling, D. E., Gauthier, L. D., Levy-Moonshine, A., Roazen, D., Shakir, K., Thibault, J., Chandran, S., Whelan, C., Lek, M., Gabriel, S., Daly, M. J., Neale, B., MacArthur, D. G. & Banks, E. Scaling accurate genetic variant discovery to tens of thousands of samples. *bioRxiv* 201178 (2018). Accessed August 20, 2022. doi:10.1101/201178

45. Koboldt, D. C., Chen, K., Wylie, T., Larson, D. E., McLellan, M. D., Mardis, E. R., Weinstock, G. M., Wilson, R. K. & Ding, L. VarScan: variant detection in massively parallel sequencing of individual and pooled samples. *Bioinformatics* **25,** 2283–2285 (2009).

46. Cingolani, P., Platts, A., Wang, L. L., Coon, M., Nguyen, T., Wang, L., Land, S. J., Lu, X. & Ruden, D. M. A program for annotating and predicting the effects of single nucleotide polymorphisms, SnpEff: SNPs in the genome of *Drosophila melanogaster* strain w1118; iso-2; iso-3. *Fly (Austin).* **6,** 80–92 (2012).

47. Kopylova, E., Noé, L. & Touzet, H. SortMeRNA: fast and accurate filtering of ribosomal RNAs

in metatranscriptomic data. *Bioinformatics* **28,** 3211–3217 (2012).

48. Patro, R., Duggal, G., Love, M. I., Irizarry, R. A. & Kingsford, C. Salmon provides fast and bias-aware quantification of transcript expression. *Nat. Methods 2017 144* **14,** 417–419 (2017).

49. Love, M. I., Anders, S. & Huber, W. Differential analysis of count data - the DESeq2 package. *Genome Biol.* **15,** 550 (2014).

50. Langmead, B. & Salzberg, S. L. Fast gapped-read alignment with Bowtie 2. *Nat. Methods 2012 94* **9,** 357–359 (2012).

51. Ramírez, F., Ryan, D. P., Grüning, B., Bhardwaj, V., Kilpert, F., Richter, A. S., Heyne, S., Dündar, F. & Manke, T. deepTools2: a next generation web server for deep-sequencing data analysis. *Nucleic Acids Res.* **44,** W160–W165 (2016).

52. Zhang, Y., Liu, T., Meyer, C. A., Eeckhoute, J., Johnson, D. S., Bernstein, B. E., Nussbaum, C., Myers, R. M., Brown, M., Li, W. & Shirley, X. S. Model-based analysis of ChIP-Seq (MACS). *Genome Biol.* **9,** 1–9 (2008).

53. Rappsilber, J., Mann, M. & Ishihama, Y. Protocol for micro-purification, enrichment, pre-fractionation and storage of peptides for proteomics using StageTips. *Nat. Protoc. 2007 28* **2,** 1896–1906 (2007).

54. Kelstrup, C. D., Young, C., Lavallee, R., Nielsen, M. L. & Olsen, J. V. Optimized fast and sensitive acquisition methods for shotgun proteomics on a quadrupole orbitrap mass spectrometer. *J. Proteome Res.* **11,** 3487–3497 (2012).

55. Bekker-Jensen, D. B., Martínez-Val, A., Steigerwald, S., Rüther, P., Fort, K. L., Arrey, T. N., Harder, A., Makarov, A. & Olsen, J. V. A compact quadrupole-orbitrap mass spectrometer with FAIMS interface improves proteome coverage in short LC gradients. *Mol. Cell. Proteomics* **19,** 716–729 (2020).

56. Olsen, J. V., Macek, B., Lange, O., Makarov, A., Horning, S. & Mann, M. Higher-energy C-trap dissociation for peptide modification analysis. *Nat. Methods 2007 49* **4,** 709–712 (2007).

57. Cox, J. & Mann, M. MaxQuant enables high peptide identification rates, individualized p.p.b.-range mass accuracies and proteome-wide protein quantification. *Nat. Biotechnol.* **26,** 1367–1372 (2008).

58. Cox, J., Neuhauser, N., Michalski, A., Scheltema, R. A., Olsen, J. V. & Mann, M. Andromeda: a peptide search engine integrated into the MaxQuant environment. *J. Proteome Res.* **10,** 1794–1805 (2011).

59. Elias, J. E. & Gygi, S. P. Target-decoy search strategy for increased confidence in large-scale protein identifications by mass spectrometry. *Nat. Methods 2007 43* **4,** 207–214 (2007).

60. Tyanova, S., Temu, T., Sinitcyn, P., Carlson, A., Hein, M. Y., Geiger, T., Mann, M. & Cox, J. The Perseus computational platform for comprehensive analysis of (prote)omics data. *Nat. Methods 2016 139* **13,** 731–740 (2016).
